# Expansion of CD101⁻ neutrophils drives susceptibility to hyperyersiniabactin-producing *Yersinia* infection in hereditary hemochromatotic hosts

**DOI:** 10.1128/iai.00691-25

**Published:** 2026-06-12

**Authors:** Shreya Das, Saugata Majumder, Mohd Saqib, McKenzie Van der Veer, Bossardi Ramos Ramon, Akaki Tsilosani, Wenzheng Zhang, Qi Yang, Sridar V. Chittur, Wei Sun

**Affiliations:** 1 Department of Immunology and Microbial Disease, Albany Medical College 1092, Albany, New York, USA; 2 Department of Molecular and Cellular Physiology, Albany Medical College 1092, Albany, New York, USA; 3 Department of Regenerative & Cancer Cell Biology, Albany Medical College 1092, Albany, New York, USA; 4 Rutgers Institute for Translational Medicine and Science, Child Health Institute of New Jersey, The State University of New Jersey, New Brunswick, New Jersey, USA; 5 Center for Functional Genomics, University at Albany-State University of New York, Rensselaer, New York, USA; University of Pennsylvania School of Veterinary Medicine, Philadelphia, Pennsylvania, USA

**Keywords:** neutrophils, immature neutrophils, sepsis, hemochromatosis, *Yersinia*, type 1 IFN

## Abstract

Hereditary hemochromatosis (HH) increases susceptibility to bacterial infections. In our previous study, an oral *Yersinia pseudotuberculosis* Δ*fur* mutant (Δ*fur*) infection led to intestinal barrier disruption and acute mortality in HH (*Hfe^−/−^*) mice. However, the systemic features of this fulminant infection are incompletely characterized. Here, HH mice infected with a hyperyersiniabactin-producing Δ*fur* rapidly develop sepsis symptoms, marked by an influx of immature CD101^−^ neutrophils with impaired bactericidal capacity and heightened inflammation, whereas infected wild-type mice harbor functional mature CD101^+^ neutrophils. This phenotype is partially recapitulated by the hyperyersiniabactin-producing clinical isolate, *Y. enterocolitica* WA strain. We further show that type I interferon (IFN-I) signaling impairs neutrophil bactericidal capacity, increasing bacterial burden, disrupting barrier disruption, and promoting emergency granulopoiesis, resulting in massive recruitment of CD101^−^ neutrophils in Δ*fur*-infected HH mice. Blocking IFN-I signaling restores neutrophil function, reduces bacterial loads, limits neutrophil recruitment, promotes a shift toward CD101^+^ neutrophils, preserves barrier integrity, mitigates hyperinflammation, and improves survival. These findings reveal that in HH mice infected with high-yersiniabactin producing *Yersinia* species, immature CD101^−^ neutrophils act as key mediators of sepsis, with IFN-I signaling as an essential regulator of this pathogenic response.

## INTRODUCTION

Hereditary hemochromatosis (HH), an inherited disease attributed to the mutated genes encoding HFE (homeostatic iron regulator), transferrin receptor 2, hemojuvelin, or hepcidin itself, results in hepcidin reduction or deficiency (1), eventually leading to excessive iron load in multiple organs (2). Among those, HH caused by the *Hfe^C282Y/C284Y^* mutation is one of the most common disorders in the United States, primarily affecting individuals of Caucasian descent (3). Excess iron impairs immune function and increases susceptibility to bacterial infections (4). Clinical reports have shown that individuals with HH are prone to infections by various bacterial pathogens, including *Yersinia* (5, 6), *Vibrio vulnificus* (7, 8), and others (9), leading to serious complications, even sepsis. Individuals with HH (C282Y homozygotes) had a higher risk of infection, along with a markedly increased risk of sepsis (9, 10). Therefore, sepsis and iron overload are tightly coupled: excess iron not only fuels pathogen proliferation but also weakens host defenses (11).

Our previous study reported that a *Yersinia pseudotuberculosis* Δ*fur* mutant (termed Δ*fur*), a hyperyersiniabactin producing mutant, exhibited high virulence in HH mice following oral infection, in contrast to its marked attenuation in wild-type C57BL/6 mice. Δ*fur* rapidly breached the intestinal barrier, disseminated systemically, and triggered a surge of inflammatory cytokines, culminating in acute mortality within 7–8 days post-infection (dpi) (12). Observations in Δ*fur*-infected HH mice strongly indicate sepsis symptoms. However, the systemic impacts of Δ*fur* causing hypersusceptibility in HH mice have not been evaluated yet.

Anti-Ly6G treatment markedly improved survival in Δ*fur*-infected HH mice (12), implicating neutrophils as a key player in driving infection severity in iron-overloaded hosts. Neutrophils serve as a double-edged sword during sepsis, critical for pathogen clearance yet capable of amplifying systemic inflammation. The presence of neutrophil heterogeneity determines disease outcomes. Low-density neutrophils, including granulocytic myeloid-derived suppressor cells that suppress T cell functions, promote immune dysfunction, impair microbial clearance, and worsen clinical outcomes in sepsis (13, 14). CD101, an immunoglobulin superfamily member containing the conserved glutamic acid-tryptophan-isoleucine (EWI) motif, marks and regulates neutrophil maturation and is linked to aberrant myelopoiesis and inflammation (15, 16). However, the role of neutrophils in modulating Δ*fur* infection in hemochromatotic hosts and the relevance of CD101 in this context have not been defined. Since bacterial infections are the leading cause of sepsis (17) and iron overload increases susceptibility (9, 10), dissecting how excess iron alters neutrophil features is essential for developing effective strategies to mitigate the severe outcomes.

In this study, we demonstrate that the fulminant systemic infection in Δ*fur*-infected HH mice is associated with infection-induced disseminated intravascular coagulation (DIC), hyperinflammation, and multiorgan injury (including liver and kidneys). A similar exacerbation was observed with a hyperyersiniabactin-producing clinical strain, *Y. enterocolitica* (Ye) WA. Neutrophils emerged as the primary mediators of pathology. Using complementary *ex vivo* assays and pharmacological interventions, we identified two distinct neutrophil subsets, defined by CD101 expression, that differentially regulate inflammatory and antimicrobial responses during sepsis. Mechanistically, the type I interferon (IFN-I) signaling pathways were found to primarily govern these subsets, revealing a mechanistic link between iron overload, neutrophil dysregulation, and sepsis outcomes in HH hosts infected with hyperyersiniabactin-producing *Yersinia* strains.

## RESULTS

### ∆*fur* infection induces hyperinflammation and provokes sepsis symptoms in HH mice

**Fig 1:**
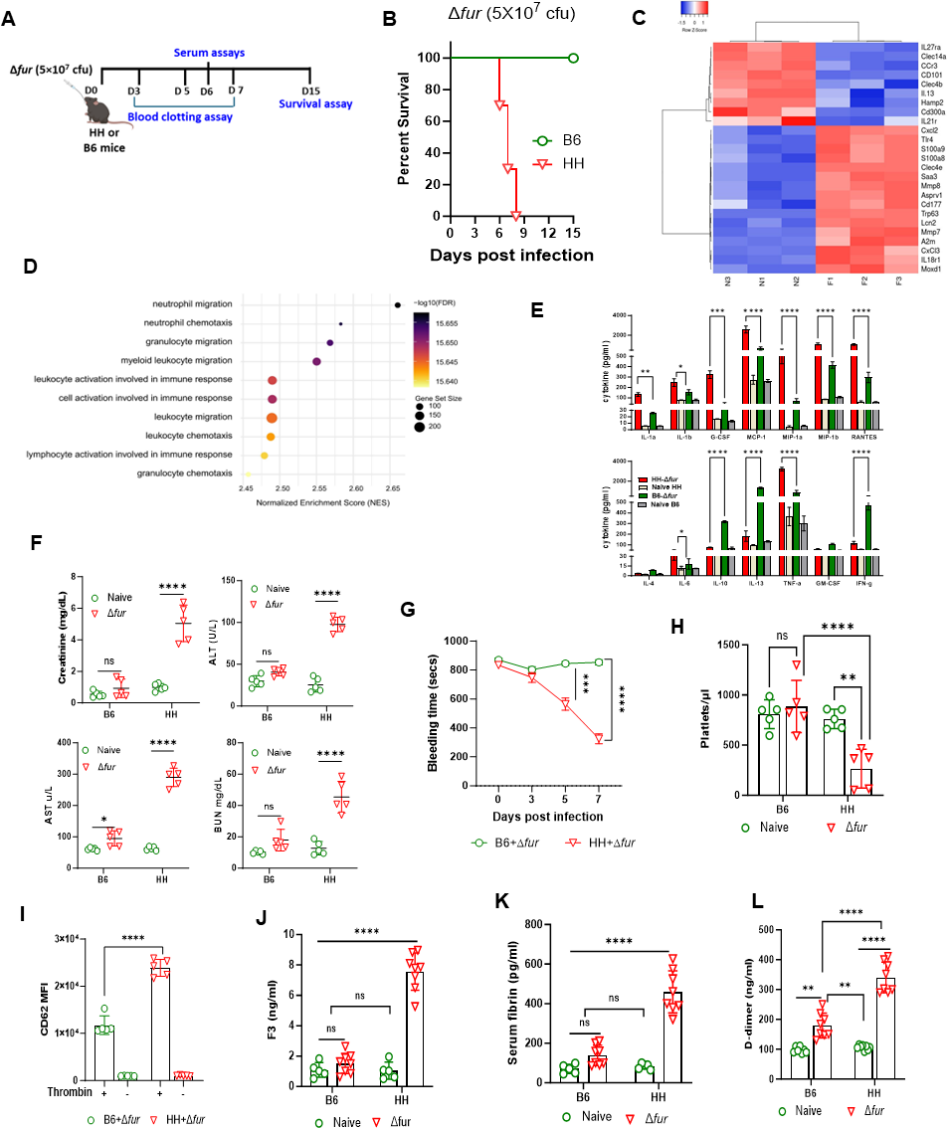
Δ*fur* infection promotes hyperinflammation and severe systemic infection characterized by DIC in HH mice. (**A**) Schema for ∆*fur* infection of B6 and HH mice, assay time points, and survival period. (**B**) Survival of ∆*fur-*infected B6 and HH mice (*n* = 10/group). (**C**) Heatmap of selected host transcripts obtained from whole transcriptome RNA-seq analysis of liver homogenates from naïve and Δ*fur*-infected HH mice at 6 dpi (*n* = 3 per group). The heatmap was constructed based on DESeq2 analysis; color coding reflects rlog-transformed read count values. (**D**) Gene Ontology functional clustering of upregulated genes associated with biological processes; the most significantly enriched categories are shown. (**E**) Cytokine analysis of liver homogenates from naïve and Δ*fur*-infected HH and B6 mice at 6 dpi (*n* = 3 per group). (**F**) Serum creatinine, ALT, AST, and BUN levels of ∆*fur-*infected HH and B6 mice at 6 dpi. Serum samples from naïve B6 or HH mice served as uninfected controls. (**G**) Coagulation assay. Time to cease bleeding in response to tail injury from ∆*fur-*infected B6 and HH mice (*n* = 6/group). (**H**) Platelet counts in mice at 6 dpi. (**I**) Mean fluorescence intensity of CD62P for platelets collected from ∆*fur-*infected B6 and HH mice at 6 dpi treated with/without thrombin. (**J–L**) The levels of blood markers of DIC. (**J**) F3, (**K**) fibrin, and (**L**) D-dimer from ∆*fur-*infected B6 and HH mice at 6 dpi. Serum from naïve HH and B6 mice served as uninfected controls. The log-rank (Mantel-Cox) test was used for survival analysis in (**A**). For all other graphs (**E–L**), statistical analyses of comparisons of data among groups were performed with the or two-way ANOVA with the Tukey post-hoc test. Data are presented as the mean ± standard deviation (ns, no significance; *P < 0.05; **P < 0.01; ***P < 0.001; ****P < 0.0001).

Our previous study demonstrated that infection with the Δ*fur* strain resulted in rapid disruption of the intestinal barrier in HH mice, leading to hyperinflammation and acute mortality (12). Confirming our previous observation, HH mice infected with Δ*fur* succumbed within 8 dpi, whereas all B6 mice survived (Fig. 1A and B). Also, transcriptomic analysis of livers from Δ*fur*-infected naïve and HH mice revealed significant upregulation of genes associated with liver injury (Moxd1, A2m), inflammation, neutrophil recruitment (Cxcl2, Cxcl3, S100a8, S100a9, Lcn2), and metal scavenging/fibrinolysis (Mmp8, Mmp9, Saa3) (Fig. 1C). Gene ontology (GO) analysis highlighted enrichment of pathways related to neutrophil migration, chemotaxis, and inflammatory responses (Fig. 1D). Accordingly, proinflammatory cytokines, including IL-1α, IL-1β, MCP-1, MIP-1α, MIP-1β, and G-CSF, were significantly elevated in Δ*fur*-HH livers (Fig. 1E), indicating hyperinflammation. We next examined the systemic impacts of Δ*fur* infection in HH mice (Fig. 1A). By 6 dpi, infected HH mice developed acute liver and kidney injuries, as evidenced by significantly elevated serum levels of creatinine, alanine aminotransferase (ALT), aspartate aminotransferase (AST), and blood urea nitrogen (BUN) (Fig. 1F), along with pronounced hepatic granulocyte infiltration and granuloma formation (Fig. S1A). Expression of fibrosis and injury markers, including alpha smooth muscle actin (α-SMA), collagen, fibronectin, and vimentin, was markedly increased in both liver (Fig. S1B) and kidney (Fig. S1C), indicating multiorgan damage and sepsis-like pathology. Furthermore, we measured several sepsis-associated indices, including coagulation and platelet activity in Δ*fur*-infected HH mice. Compared with infected B6 mice, infected HH mice exhibited shortened clotting times (Fig. 1G), reduced platelet counts (Fig. 1H), and enhanced platelet activation (CD62P^+^) (Fig. 1I). Elevated levels of D-dimer, tissue factor (F3), and fibrin levels (Fig. 1J through L) further confirmed disseminated intravascular coagulation, a hallmark of sepsis (18, 19). Collectively, these findings demonstrate that Δ*fur* infection triggers systemic hyperinflammation and sepsis, leading to acute multiorgan failure and death in HH hosts.

To determine whether ∆*fur* hypervirulence was associated with host iron levels, HH mice were treated intraperitoneally with the iron chelator deferoxamine (3 mg/dose, DFO) daily from −5 to 0 dpi (Fig. S2). DFO treatment significantly reduced systemic iron levels compared to vehicle-treated controls (Fig. S2A). Following oral infection with ∆*fur*, DFO-treated HH mice showed prolonged survival up to 15 days (Fig. S2B). At 6 dpi, DFO treatment modestly improved sepsis and DIC markers compared to vehicle controls (Fig. S2C through E). In addition, anticoagulant treatment with rivaroxaban, a factor Xa inhibitor (Fig. S3A), slightly reduced coagulation, and liver and kidney injuries in Δ*fur*-infected HH mice (Fig. S3B through D) but not the systemic bacterial load and survival (Fig. S3E and F). These findings indicate that Δ*fur* infection in HH mice triggers irreversible immune dysregulation that drives DIC and sepsis, resulting in severe pathology.

HH causes progressive, age-dependent iron accumulation in vital organs, leading to increased risk of infection (20). *Y. enterocolitica* serotype O:8 represents a highly virulent lineage and is prevalent in the U.S. (21). Strains O:8/1B produce high levels of yersiniabactin, promoting replication under iron-restricted cues (22), and are clinically associated with severe infections (23). Here, Ye WA, an O:8/1B strain isolated from a patient (24), was used to assess infection in age-differentiated HH mice and recapitulate the clinical relevance of the Δ*fur* findings. Compared to age-matched B6 controls, HH mice were highly susceptible to Ye WA in an age-dependent manner (Fig. S3G). Like Δ*fur* Δ*irp2* Yptb PB1+ strain (12), the Δ*irp2* Ye WA strain, lacking yersiniabactin, was fully avirulent in HH mice (Fig. S3G). These findings suggest that host iron levels and high yersiniabactin production determine *Yersinia* disease severity.

### Excessive neutrophil recruitment amplifies ∆*fur*-induced pathology in HH mice

**Fig 2:**
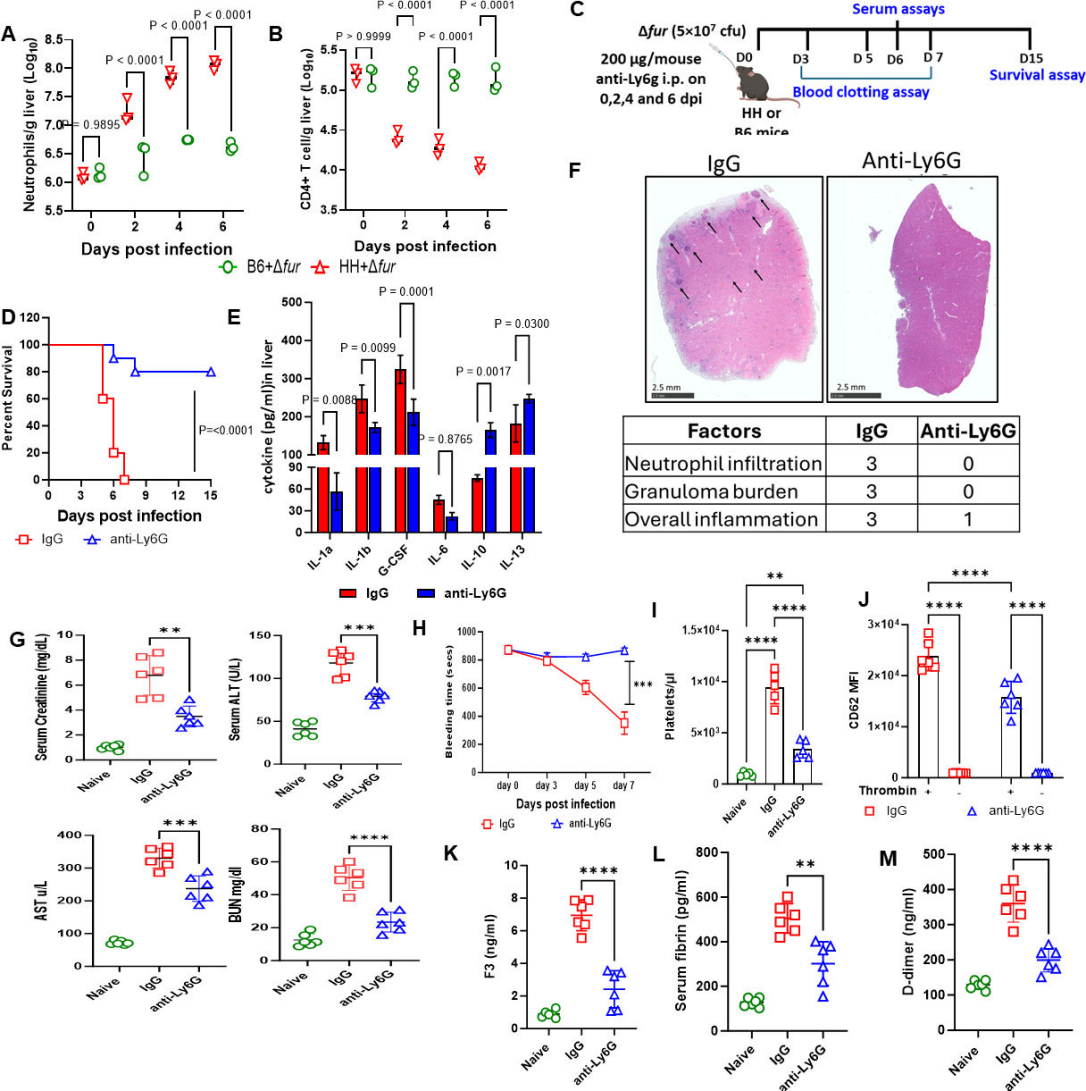
Neutrophils play a critical role in exacerbating ∆*fur*-induced pathology in HH mice. (**A and B**) Flow cytometric quantification of immune cell populations in Δ*fur*-infected HH and B6 mice at 0, 3, 5, and 7 dpi (*n* = 3 per group): (**A**) neutrophils and (**B**) CD4^+^ T cells. (**C**) Schematic illustrating neutrophil depletion in Δ*fur*-infected HH mice. (**D**) Survival of ∆*fur-*infected HH mice treated with anti-Ly6G antibodies or IgG2a isotype control (*n* = 10/group). (**E**) Cytokine levels in liver homogenates at 6 dpi from Δ*fur*-infected HH mice treated as mentioned above (*n* = 4/group). (**F**) Representative hematoxylin and eosin-stained liver histopathology images from the same mice and histopathological scores. (**G**) Serum levels of creatinine, ALT, AST, and BUN were measured by ELISA. (**H**) Time to cessation of bleeding. (**I**) Platelet counts at 6 dpi in Δ*fur*-infected HH mice treated with anti-Ly6G or IgG. (**J**) Mean fluorescence intensity of CD62P on platelets treated with or without thrombin stimulation at 6 dpi. (**K–M**) Serum levels of (**K**) F3, (**L**) fibrin, and (**M**) D-dimer in Δ*fur*-infected HH mice treated with anti-Ly6G or IgG. Serum from naïve mice served as uninfected controls. For all panels, unless otherwise mentioned, each symbol represents data from an individual mouse. Statistical analyses of comparisons of data among groups were performed with an unpaired *t*-test using a parametric test (**G, K–M**), one-way ANOVA/univariate (**I and J**), or two-way ANOVA with the Tukey post-hoc test (A, B, E, and H). The log-rank (Mantel-Cox) test was used for survival analysis (**D**). Data are presented as the mean ± standard deviation (ns, no significance; *P < 0.05; **P < 0.01; ***P < 0.001; ****P < 0.0001).

To assess hepatic cellular responses, live CD45^+^ cells were gated from the liver single-cell suspension as described in Fig. S4A. Evaluation of cellular influx revealed that ∆*fur*-infected HH mice exhibited a substantial increase (27-fold ± 7, *P* < 0.0001) in neutrophil (CD11b^+^Ly6G^+^) infiltration in the liver compared to infected B6 mice at 6 dpi (Fig. 2A). Similar increases in neutrophil recruitment were also observed in the spleen and blood of ∆*fur*-infected HH mice (Fig. S4B). In contrast, neutrophil numbers in the bone marrow (BM) of ∆*fur*-infected HH mice were significantly lower than in infected B6 mice (Fig. S4B), suggesting enhanced mobilization or depletion from the bone marrow. Macrophages in ∆*fur*-infected HH mice peaked at 3 dpi but reduced thereafter and remained only moderately higher than B6 levels at 6 dpi (Fig. S4C), while monocyte counts remained comparable between strains (Fig. S4D). Interestingly, liver CD4^+^ T cells declined sharply in HH mice, contrasting with stable T cell counts in B6 mice (Fig. 2B). Consequently, the neutrophil-to-T cell ratio, a hallmark of sepsis and septic shock (25, 26), elevated dramatically, reaching 10,711.99 (± 2,391, *P* < 0.0001) in HH mice compared to 35.11 (± 11.18, *P* = 0.8952) in B6 mice at 6 dpi, indicating profound immune imbalance (Fig. 2A and B).

Consistent to our earlier study (12), neutrophil depletion via anti-Ly6G antibodies markedly improved disease outcomes, resulting in 80% survival for ∆*fur-*infected HH mice (Fig. 2C and D). Anti-Ly6G treatment in ∆*fur*-infected HH mice significantly reduced hepatic cytokine storm (Fig. 2E), mitigated granulomatous lesions and liver injury (Fig. 2F, *P* < 0.0001), and lowered serum creatinine, AST, ALT, and BUN (Fig. 2G). It also prolonged clotting time, restored platelet counts, and reduced platelet activation, plasma F3, fibrin, and D-dimer levels (Fig. 2H through M). Similar observations were also observed in the case of Ye WA infection, wherein treatment of anti-Ly6G antibodies improved disease and resulted in 80% survival compared to IgG control-treated mice (Fig. S5A through E). These findings suggest uncontrolled neutrophilia primarily drives disease exacerbation in ∆*fur*-infected HH mice.

### Differential CD101 expression on neutrophils correlates with mouse susceptibility to ∆*fur*

**Fig 3:**
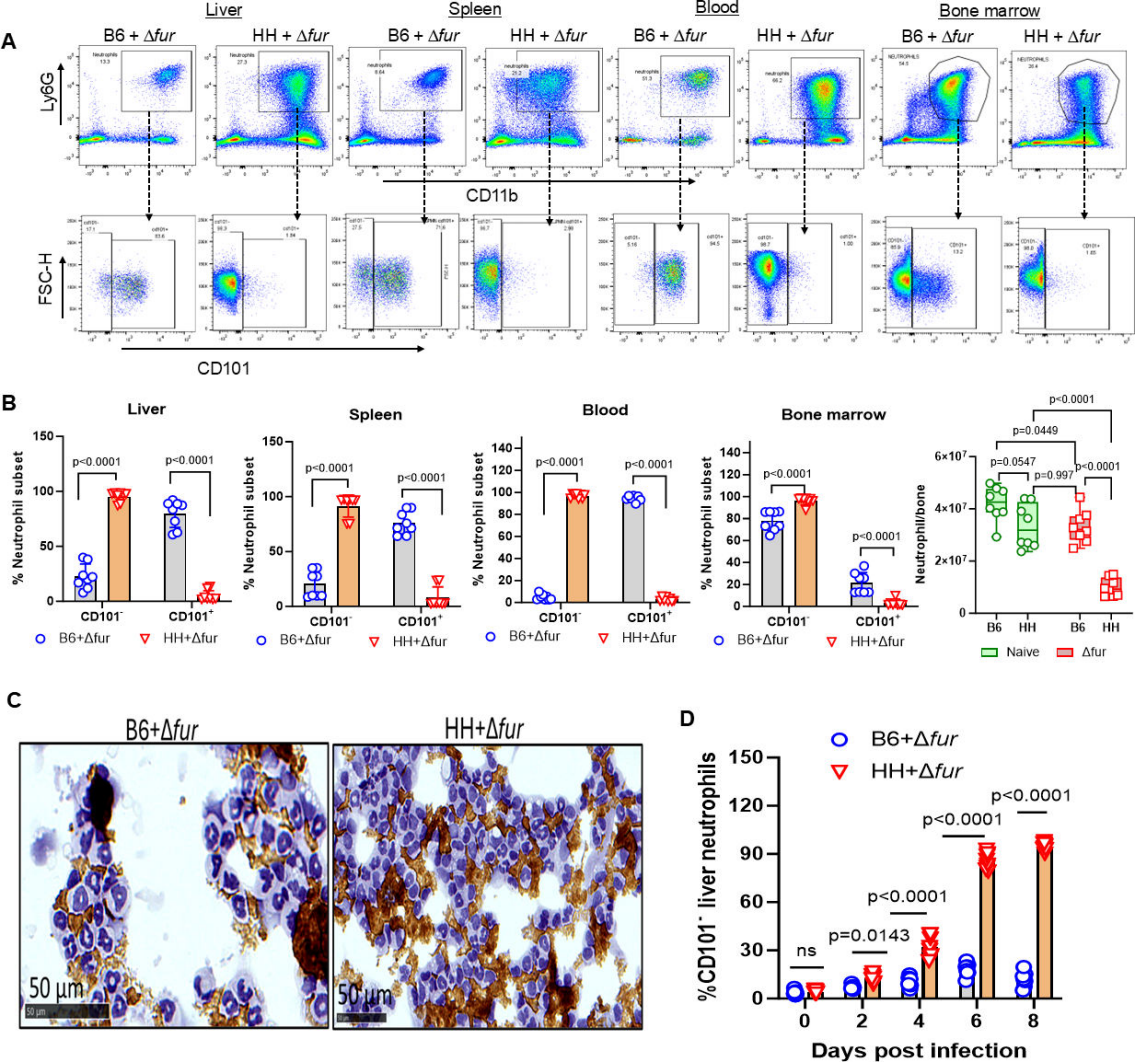
Susceptibility to ∆*fur* infection correlates with differential CD101 expression on neutrophils. (**A**) Representative fluorescence-activated cell sorting plots showing CD101 expression on neutrophils in various organs of Δ*fur*-infected B6 and HH mice at 6 dpi (*n* = 8 per group). (**B**) Quantitative plots showing systemic distribution of CD101^+^ and CD101^−^ neutrophil subsets in Δ*fur*-infected B6 and HH mice at 6 dpi. (**C**) Representative hematoxylin and eosin-stained images of mag sorted neutrophils from the blood of Δ*fur*-infected HH and B6 mice at 6 dpi (*n* = 3 per group). (**D**) Kinetics of CD101^−^ neutrophil infiltration into the liver of Δ*fur*-infected HH and B6 mice at the indicated time points (*n* = 8 per group). Statistical analyses of comparisons of data among groups were performed with two-way ANOVA with the Tukey post-hoc test. Data are presented as the mean ± standard deviation (ns, no significance; *P < 0.05; **P < 0.01; ***P < 0.001; ****P < 0.0001).

The ratio of immature neutrophils to the total number of neutrophils in the blood circulation is often used as a diagnostic indicator for sepsis (27–29). Mature neutrophils are characterized by CD11b^+^Ly6G^hi^CD101^+^ cells on their surface, whereas immature neutrophils are Ly6G^lo/+^ expression and lack CD101 (27, 28). Flow cytometry analysis showed that neutrophils in the blood, liver, and spleen of ∆*fur*-infected HH mice at 6 dpi were predominantly CD11b^+^Ly6G^lo/+^, whereas infected B6 mice primarily exhibited CD11b^+^Ly6G^hi^ neutrophils (Fig. 3A). Further analysis of the CD101 expression showed that naïve HH and B6 mice had abundant CD101^+^ neutrophils in the liver and spleen while most CD101^−^ neutrophils in their bone marrow (Fig. S6A). Naïve HH mice exhibited a very modest increase in the CD101⁻ neutrophils in the liver and bone marrow compared to the naïve B6 mice, which could be due to the iron overload in HH mice. However, no alteration with respect to CD101 expression was observed in the spleen of naïve HH or B6 mice (Fig. S6A). Similar to naïve HH or B6 mice, the neutrophils in ∆*fur*-infected B6 mice liver, spleen, and blood were primarily CD101^+^ and contained a very low percentage of CD101^−^ neutrophils (Fig. 3A and B). However, ∆*fur*-infected HH mice showed a remarkably less CD101^+^ neutrophils, accompanied by a significant increase in CD101^−^ neutrophils in the liver, spleen, blood, and bone marrow compared to infected B6 mice at 6 dpi (Fig. 3A and B). In the bone marrow, both infected HH and B6 mice showed a higher proportion of CD101^−^ neutrophils than CD101^+^ neutrophils; however, infected HH mice exhibited a significantly greater expansion of the CD101^−^ subset compared to infected B6 mice (Fig. 3A and B). Similar accumulation of CD101^−^ neutrophils was also observed in Ye WA-infected HH mice (Fig. S6B). Results indicate emergency granulopoiesis, while the lower CD101^+^ neutrophil frequency in the infected HH bone marrow compared to infected B6 mice is indicative of bone marrow exhaustion and failure.

Furthermore, to evaluate the role of CD101 as a maturation marker in neutrophils, using the Neutrophil Isolation Kit (BioLegend), we isolated neutrophils from the blood of *∆fur*-infected HH and B6 mice at 6 dpi. At this point, B6 mice predominantly harbored CD101^+^ neutrophils, whereas HH mice harbored CD101^−^ neutrophils (Fig. 3A and B). Nuclear morphology assessed by hematoxylin and eosin (H&E) staining revealed that neutrophils in B6 liver majorly exhibited segmented, multilobed nuclei, characteristic of mature cells. In contrast, neutrophils from HH mice exhibited unsegmented nuclear morphologies characteristic of immature developmental stages (Fig. 3C).

Subsequently, the kinetics of CD101^−^ immature neutrophils and their association with disease severity were assessed (Fig. 3D). During the early phase of infection (0–2 dpi), CD101^−^ neutrophils comprised less than 15% ± 4% of the total liver neutrophil population, with comparable proportions (*P* = 0.0143) observed in both B6 and HH mice. By 4–6 dpi, a significant expansion (*P* < 0.0001) of this population was observed in HH mice (30.33% ± 7% at 4 dpi, 85.85% ± 5% at 6 dpi), whereas it remained at stable levels (around 10% ± 3%) in B6 mice. This trend became more pronounced by 8 dpi, when 94.76% ± 3% of liver neutrophils in HH mice were CD101^−^, compared to only 15.33% ± 5% in B6 mice (*P* < 0.0001) (Fig. 3C). The kinetics of expansion of CD101^−^ neutrophils in HH mice are strongly correlated with the onset of DIC and worsening sepsis (Fig. 1G through L). The findings indicate that ∆*fur*-induced disease exacerbation in HH mice coincides with the accumulation of CD101^−^ immature neutrophils, implicating this subset as a key driver of immunopathology and disease progression during infection.

### CD101^−^ immature neutrophils in HH mice show reduced bactericidal and enhanced proinflammatory features

**Fig 4:**
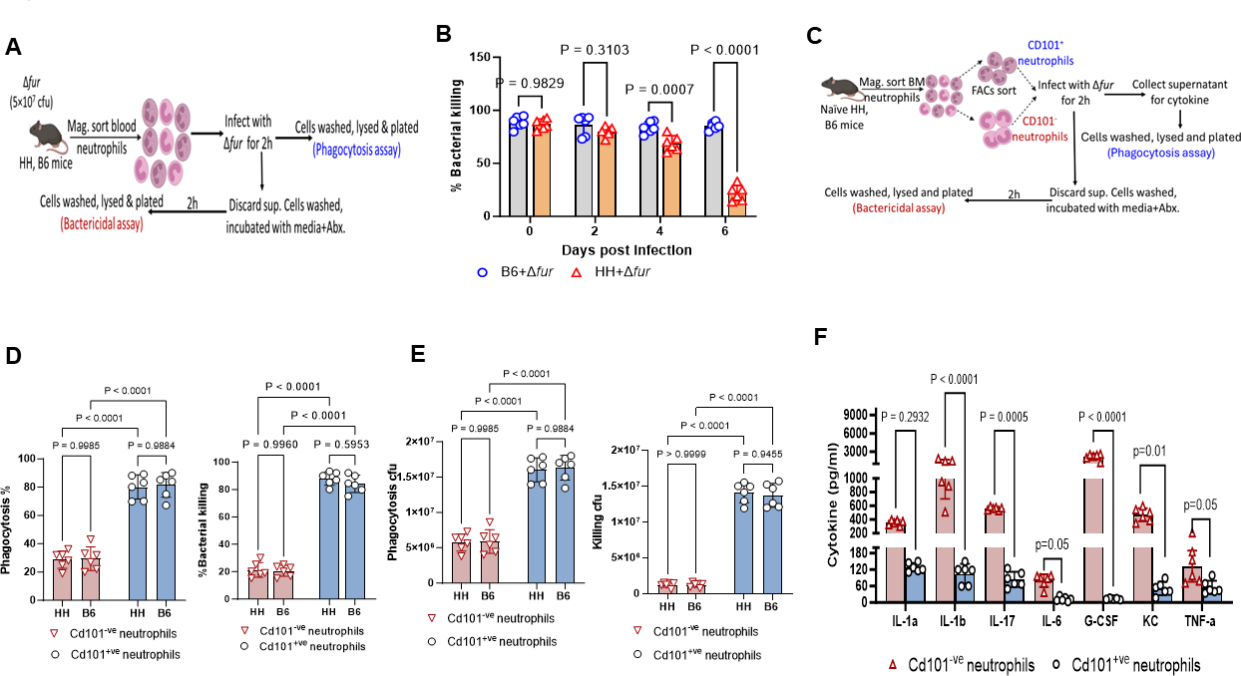
CD101^−^ neutrophil subset has reduced antimicrobial capacity and is proinflammatory. (**A**) Schematic and (**B**) kinetics of the antimicrobial activity of purified blood neutrophils from Δ*fur*-infected HH and B6 mice at the indicated time points. Magnetically sorted neutrophils from the blood of infected HH or B6 mice were infected with ∆*fur* at a multiplicity of infection of 5 as described in Materials and Methods. Bactericidal activity was calculated by comparing the colony-forming unit (CFU) counts obtained after the bactericidal assay to those from the phagocytosis assay. (**C**) Schematic for (**D**) phagocytosis percentage or bactericidal percentage (**E**) CFU count from phagocytosis or bactericidal assay (**F**) cytokine production assays using CD101^+^ and CD101^−^ neutrophils isolated from the bone marrow of naïve B6 and HH mice and infected *ex vivo* with Δ*fur* as mentioned above for 2 h. Supernatants were used for cytokine analysis, and bactericidal activity was calculated as mentioned above. For all panels, unless otherwise mentioned, each symbol represents data from an individual mouse. Statistical analyses of comparisons of data among groups were performed with two-way ANOVA with the Tukey post-hoc test. Data are presented as the mean ± standard deviation (ns, no significance; *P < 0.05; **P < 0.01; ***P < 0.001; ****P < 0.0001).

To assess the role of CD101 as a maturation marker and neutrophil function, blood neutrophils from Δ*fur*-infected B6 and HH mice were isolated by magnetic sorting at 0, 2, 4, and 6 dpi and infected *ex vivo* with Δ*fur*. Their bactericidal capacity was then measured as outlined in the experimental schema (Fig. 4A). Results showed that neutrophils from naïve HH and B6 mice at 0 and 2 dpi exhibited no significant difference in their bactericidal capacity and displayed 85.66% ± 10% bactericidal capacity, respectively. At 4 and 6 dpi, ∆*fur*-infected HH mice had mostly CD101⁻ neutrophils (Fig. 3D), and their bactericidal capacity dropped to 68.79% ± 5.3% at 4 dpi and 21.87% ± 4.9% at 6 dpi (Fig. 4B). In contrast, B6 mice predominantly had CD101^+^ neutrophils at 4 and 6 dpi (Fig. 3D) and remained at high bactericidal capacity (84.33% ± 2.3% and 83.66% ± 4%, respectively) (Fig. 4B). Furthermore, CD101^−^ and CD101^+^ neutrophils were fluorescence-activated cell sorting (FACS)-sorted from the bone marrow of naïve HH and B6 mice, respectively. Cells (4 × 10^6^ cells/well) were infected *ex vivo* with ∆*fur*, and the phagocytic and bactericidal capacity was measured as depicted in the schema (Fig. 4C). CD101^+^ neutrophils from both HH and B6 mice exhibited strong phagocytic and bactericidal capacity, whereas CD101^−^ neutrophils from both B6 and HH mice exhibited significantly reduced phagocytic and bactericidal capacity (Fig. 4D and E). Parallelly, cytokine levels in the supernatant from above *ex vivo* ∆*fur*-infected neutrophils were measured by ELISA. CD101^−^ neutrophils released significantly higher levels of IL-1β, TNF-α, and G-CSF compared to CD101^+^ neutrophils (Fig. 4F). These results indicate that CD101^−^ neutrophils have impaired bacterial killing capacity and exhibit a proinflammatory phenotype upon ∆*fur* infection.

### Systemic accumulation of CD101^−^ neutrophils is regulated by G-CSF

Excessive neutrophil migration from the BM to the bloodstream and their subsequent recruitment to infected tissues are coordinated by chemokine receptors and signaling molecules such as G-CSF (30), which may lead to increased inflammation during the early stages of sepsis (31). We reasoned that the high concentration of G-CSF in the infected HH liver (Fig. 1) could influence/regulate the mobilization and influx of the proportion of immature CD101^−^ neutrophils to the hepatic environment. Administration of recombinant G-CSF to naïve B6 mice significantly enhanced the proportion of CD101^−^ neutrophil subset among the total neutrophils in the liver and blood (Fig. S7A and B) but not in the bone marrow (Fig. S7C), indicating their migration from the bone marrow (Fig. S7A through C). Furthermore, administration of antimouse G-CSF Abs to ∆*fur*-infected HH mice significantly reduced the total number of neutrophils and proportion of CD101^−^ neutrophil subset in the liver compared to the isotype IgG-treated controls (Fig. S7D and E). The reduction in CD101^−^ neutrophils was associated with significant improvement in liver pathology in the mice receiving anti-G-CSF therapy (Fig. S7F), highlighting the importance of G-CSF signaling in the recruitment of CD101^−^ immature neutrophils. However, G-CSF neutralization only rescued 20% of the infected HH mice (Fig. S7G). Thus, these findings suggest that detrimental outcomes in ∆*fur*-infected HH mice result from a combination of multiple factors rather than by G-CSF alone.

### Dysregulated type 1 IFN signaling promotes the recruitment of CD101^−^ neutrophils in infected HH mice

**Fig 5:**
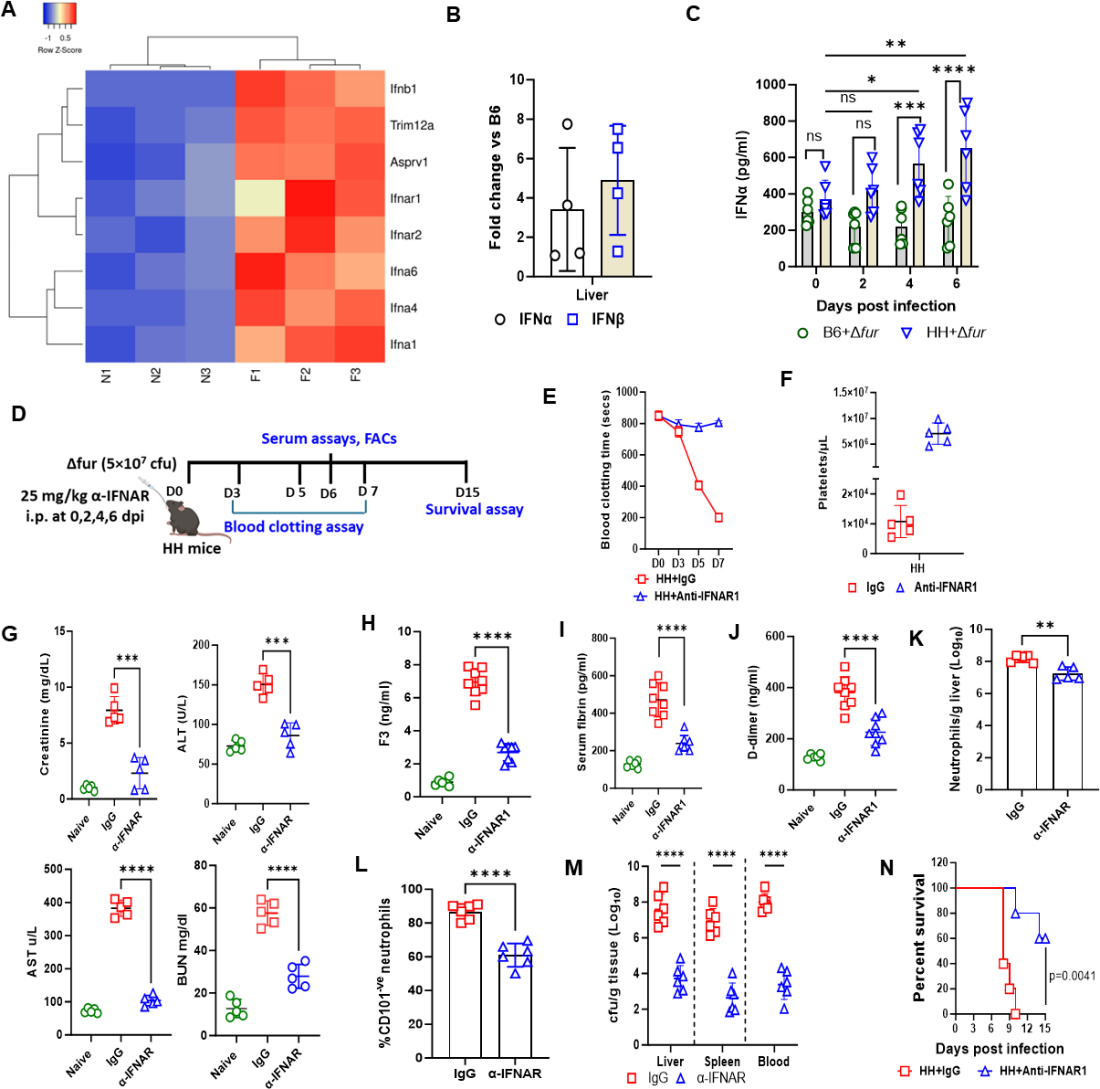
Recruitment of CD101^−^ neutrophils is regulated by type 1 IFN signaling. (**A**) Heatmap representation of selected host transcripts based on DESeq2 analysis; color coding reflects rlog-transformed read count values. (**B**) qRT-PCR quantification of IFN-α and IFN-β expression in liver homogenates of HH and B6 mice (*n* = 4) at 6 dpi. (**C**) ELISA quantification of IFN-α levels in liver homogenates of HH and B6 mice (*n* = 6) at 6 dpi. (**D**) Schematic illustrating inhibition of IFNAR signaling via anti-IFNAR antibody administration in Δ*fur*-infected HH mice. (**E**) Time to cessation of bleeding. (**F**) Platelet counts at 6 dpi in Δ*fur*-infected HH mice treated with anti-IFNAR or control rat IgG2a antibodies. (**G**) Serum levels of creatinine, ALT, AST, and BUN measured by ELISA. (**H–J**) Serum levels of (**H**) F3, (**I**) fibrin, and (**J**) D-dimer in anti-IFNAR- or IgG-treated HH mice at 6 dpi. Serum from naïve mice served as uninfected controls. (**K**) Total neutrophil counts and (**L**) proportions of CD101^+^ and CD101^−^ neutrophils in the liver of HH mice treated with anti-IFNAR or IgG at 6 dpi. (**M**) Systemic Δ*fur* load from the same mice. (**N**) Survival analysis of Δ*fur*-infected HH mice treated with anti-IFNAR or IgG antibodies. For all panels, unless otherwise mentioned, each symbol represents data from an individual mouse. Statistical analyses of comparisons of data among groups were performed with an unpaired *t*-test using a parametric test (**F–L**) or two-way ANOVA with the Tukey post-hoc test (C, E, and M). The log-rank (Mantel-Cox) test was used for survival analysis (**N**). Data are presented as the mean ± standard deviation (ns, no significance; *P < 0.05; **P < 0.01; ***P < 0.001; ****P < 0.0001).

Type I interferons are rapidly induced during infection and shape antimicrobial innate immunity (32). They also activate coagulation cascades in gram-negative sepsis, amplifying inflammation and worsening disease (33, 34). Transcriptomic profiling revealed robust induction of type I IFN signatures in ∆*fur*-infected HH livers (Fig. 5A). At 6 dpi, *Ifn-α* and *Ifn-β* expression levels were 3.41- and 4.88-fold higher in HH mice than in infected B6 controls (Fig. 5B). ELISA confirmed that, compared to IFN-α levels at 0 dpi, a sharp rise in hepatic IFN-α levels was observed at 4–6 dpi in HH but not B6 mice (Fig. 5C), correlating with progress in disease severity. Results imply that type 1 IFN may influence pathogenic CD101^−^ neutrophil function and trafficking.

Type 1 IFNs, including IFN-α and IFN-β, signal through their cell surface receptor IFN-α/βR (IFNAR) (32). Therefore, the effect of IFNAR1 neutralization on CD101^−^ neutrophil trafficking was examined in ∆*fur*-infected HH mice (Fig. 5D). Anti-IFNAR (α-IFNAR) therapy significantly ameliorated disease in ∆*fur-*infected HH mice demonstrated by improved coagulation, serum ALT, AST, creatinine, and BUN (Fig. 5E through J). Anti-IFNAR-treated mice exhibited reduced hepatic neutrophil counts with a lower proportion of the CD101^−^ subset (Fig. 5K and L), improved bacterial burden compared to isotype-treated mice (Fig. 5M), and resulted in 60% survival (Fig. 5N). Similarly, anti-IFNAR therapy in Ye WA-infected HH mice (Fig. S8A) resulted in a significant shift toward a predominance of CD101^+^ neutrophils, in contrast to the usual CD101^−^ phenotype observed in these mice (Fig. S8B), and resulted in a significant increase in survival (Fig. S8C). These results underscore the critical role of IFNAR signaling in promoting systemic infiltration of immature pathogenic CD101^−^ neutrophils, thereby exacerbating infection in HH mice.

### TLR4-mediated activation of IFN-I impairs the antimicrobial capacity of neutrophils

**Fig 6:**
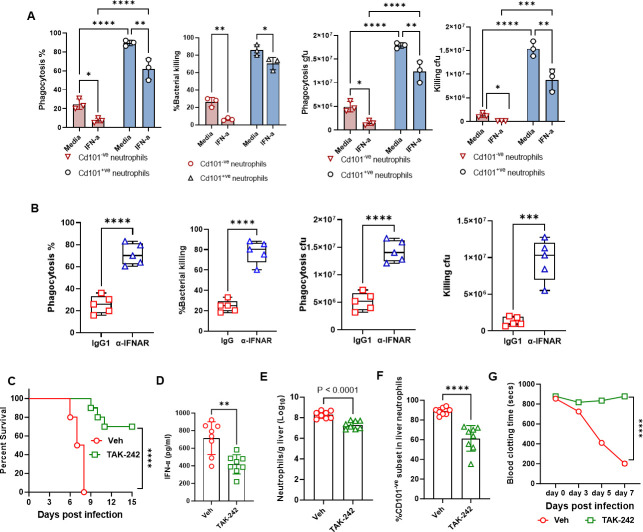
TLR4-mediated activation of IFN-1 impairs the antimicrobial capacity of neutrophils. (**A**) Phagocytic and bactericidal activity of neutrophil subsets treated *ex viv*o with IFN-α. CD101^+^ and CD101^−^ neutrophils were isolated from the bone marrow of HH mice, pretreated with IFN-α or media for 12 h, followed by infection with Δ*fur* (MOI = 5) for 2 h as described in Materials and Methods. Bactericidal activity was calculated by comparing the colony-forming unit (CFU) counts obtained after the bactericidal assay to those from the phagocytosis assay. (**B**) Phagocytic and bactericidal activity of neutrophils from Δ*fur-*infected HH mice treated with anti-IFNAR or isotype control (rat IgG2a) antibodies. (**C**) Survival analysis of Δ*fur-*infected HH mice (*n* = 10/group) treated with TLR4 agonist TAK-242 or vehicle control. (**D**) ELISA quantification of IFN-α levels in liver homogenates of Δ*fur-*infected HH mice treated with Tak-242 or vehicle control at 6 dpi. (**E**) Total neutrophil counts and (**F**) proportions of CD101^+^ and CD101^−^ neutrophils in the liver of the above mice at 6 dpi. (**G**) Time to cessation of bleeding of Δ*fur*-infected HH mice treated with TAK-242 or vehicle control. For all panels, unless otherwise mentioned, each symbol represents data from an individual mouse. Statistical analyses of comparisons of data among groups were performed with an unpaired *t*-test using a parametric test (A, C, and E–G) or two-way ANOVA with the Tukey post-hoc test (**B and H**). The log-rank (Mantel-Cox) test was used for survival analysis (**D**). Data are presented as the mean ± standard deviation (ns, no significance; *P < 0.05; **P < 0.01; ***P < 0.001; ****P < 0.0001).

To evaluate whether type 1 IFN directly impairs the phagocytic and antimicrobial capacity of neutrophils, total BM neutrophils or CD101^−^ and CD101^+^ BM neutrophils from naïve B6 mice were treated with IFN-α and evaluated for the phagocytic and bactericidal capacity of ∆*fur*. IFN-α pre-stimulation reduced ∆*fur* phagocytic and bactericidal capacity by total neutrophils (Fig. S8D) and both subsets (Fig. 6A), indicating a general mechanism unrelated to CD101 expression. Furthermore, anti-IFNAR therapy in ∆*fur-*infected HH mice significantly improved the phagocytic and bactericidal capacity of neutrophils (Fig. 6B), indicating the crucial role played by IFN-I in modulating the phagocytic and antimicrobial capacity of neutrophils.

Gram-negative bacteria or lipopolysaccharide (LPS) can stimulate the expression of type 1 IFNs through toll-like receptor 4 (TLR4) and toll/interleukine -1 (TIR)-domain-containing adapter-inducing interferon-β, an adaptor molecule downstream of TLR4 (35). To identify the source of type 1 IFN signaling, ∆*fur*-infected HH mice were treated with TAK-242, a TLR4 antagonist that selectively binds to TLR4 over other TLRs. Treatment with TAK-242 resulted in 70% survival and a significant reduction in IFN-α levels (Fig. 6C and D). Also, TAK-242 treatment significantly reduced the total neutrophil count and the proportion of the CD101^−^ neutrophil subset in the liver while also prolonging blood clotting time (Fig. 6E through G). Since Yptb LPS is a potent activator of TLR4 (36), we further assessed whether alterations in bacterial LPS affect IFN-I production. HH mice were infected with the Yptb Δ*fur* Δ*lpxP* Δ*lpxL* mutant, which lacks TLR4-active LPS but retains high yersiniabactin production. This infection markedly reduced IFN-α levels and improved survival to 80% (Fig. S8E and F). The decline in IFN-α correlated with reduced total neutrophils and a lower proportion of pathogenic CD101^−^ subsets (Fig. S8G and H), indicating that TLR4-dependent endotoxin sensing triggers IFN-α-mediated recruitment of immature neutrophils in HH hosts. Furthermore, infection of HH mice with Δ*fur* Δ*lpxP* Δ*lpxL* mutant supplemented with LPS (20 µg in 200 µL PBS/mouse, oral gavage) at 0 and 7 dpi recapitulated ∆*fur* infection (Fig. 1B) and resulted in complete death within 8–12 dpi (Fig. S8F). The control group of HH mice receiving only LPS caused no mice death (Fig. S8F). Similar outcomes were observed with the Δ*fur* Δ*irp2* mutant, which is deficient in yersiniabactin synthesis and retains intact LPS activation (Fig. S8E through H). These findings suggest that TLR4 activation, together with hyperyersiniabactin production, amplifies IFN-I signaling. Enhanced IFN-I signaling promotes intestinal barrier disruption, facilitates bacterial dissemination, and drives robust expansion of CD101^−^ neutrophils, ultimately leading to mortality.

### Type I IFN dysregulation compromises intestinal integrity and promotes systemic infection

**Fig 7:**
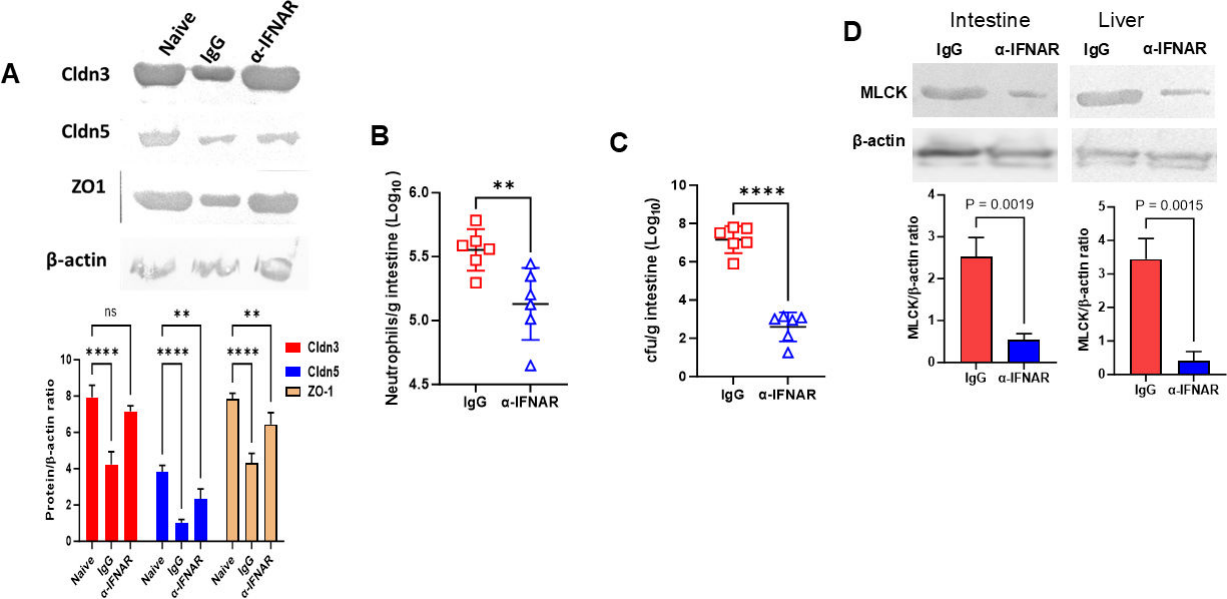
Type I IFN dysregulation compromises intestinal integrity. (**A**) Representative immunoblot analysis and quantification of protein levels from small intestine homogenates from naïve HH mice and Δ*fur*-infected HH mice (*n* = 3/group) treated with anti-IFNAR antibodies or IgG2a isotype control at 6 dpi. β-Actin was used as a loading control. (**B**) Quantitative plot for neutrophil numbers in the intestine of Δ*fur-*infected HH mice (*n* = 6/group) treated with anti-IFNAR antibodies or rat IgG2a control at 6 dpi. (**C**) Bacterial load in the intestine of the same mice. (**D**) Representative immunoblot analysis of MLCK expression and quantification of MLCK protein in intestinal homogenates of HH mice treated with anti-IFNAR antibodies or vehicle control at 6 dpi (*n* = 3/group). β-Actin was used as a loading control. The protein levels of the (**A**) tight junction proteins or (**D**) MLCK in the small intestine of Δ*fur-*infected HH mice treated with anti-IFNAR antibodies or vehicle control (*n* = 3) were quantified by ImageJ software and normalized to the level of β-actin. For all panels, unless otherwise mentioned, each symbol represents data from an individual mouse. Statistical analyses of comparisons of data among groups were performed with an unpaired *t*-test using a parametric test (**B–D**) or two-way ANOVA with the Tukey post-hoc test (**A**). Data are presented as the mean ± standard deviation (ns, no significance; *P < 0.05; **P < 0.01; ***P < 0.001; ****P < 0.0001).

Since type 1 IFN impairs phagocytic and killing capacity of neutrophils (Fig. 6A and B; Fig. S8D), we explored whether modulating IFNAR signaling could potentially reduce ∆*fur*-induced epithelial damage by affecting ∆*fur* bacterial load in the gut. Moreover, compromised bacterial killing by neutrophils could additionally promote ∆*fur* proliferation in the gut, further damaging the intestinal epithelium through LPS, yersiniabactin, and other factors. Reiterating our earlier study (12), ∆*fur*-infected HH mice resulted in enhanced disruption in the tight junction (TJ) barrier as indicated by reduced expressions of TJ barrier proteins claudin-3, claudin-5, and ZO-1 (Fig. 7A). In contrast, ∆*fur*-infected HH mice treated with anti-IFNAR antibodies displayed increased expression of TJ barrier proteins, indicating restoration of the epithelial barrier integrity. Furthermore, treatment of ∆*fur*-infected HH mice with anti-IFNAR Abs reduced neutrophil infiltration and decreased bacterial loads in the gut (Fig. 7B and C). Previously, we showed that in ∆*fur*-infected HH mice, inhibition of MLCK-mediated myosin light chain phosphorylation preserved tight junction integrity, limited bacterial dissemination, and improved survival (12). Here, treatment with anti-IFNAR Abs significantly reduced MLCK expression in the intestine and liver tissues of ∆*fur*-infected HH mice (Fig. 7D). These findings suggest that dysregulated IFNAR signaling in ∆*fur*-infected HH mice drives barrier disruption and promotes bacterial dissemination.

## DISCUSSION

Consistent with previous reports (37, 38), iron overload caused by hepcidin deficiency promotes systemic bacterial dissemination and increases the risk of sepsis. In HH (*Hfe^−/−^*) mice, ∆*fur* infection caused fulminant disease with hypercoagulation, multiorgan failure, and rapid death. A clinical Ye WA strain produced similar outcomes, mirroring the increased risk of severe infection and sepsis in hemochromatotic patients (5, 8, 10) and underscoring the study’s clinical relevance. Both Δ*fur*Δ*irp2* Yptb PB1+ (Fig. S8F) and Δ*irp2* Ye WA (Fig. S3G), Ybt-deficient strains, were fully attenuated in HH mice. The findings suggest that Ybt is a key driver of lethal infection and inflammation in HH mice and may play a similar role in other types of iron overload. In addition, high Ybt-producing *E. coli* exhibits enhanced virulence in animal models compared with the corresponding Ytb-ablating *E. coli* (39, 40), suggesting a broader role of Ybt in bacterial pathogenicity.

Interestingly, Ye Ruokola/71 (O:9), a clinical, non-Ybt-producing isolate, was avirulent in both 8-week-old HH and B6 mice (unpublished data), while it was lethal in iron-overloaded hepcidin knockout (HKO) mice (41). The reason is that HKO mice accumulate excess iron in tissues shortly after birth, whereas 8-week-old HH mice show only mildly increased iron levels (42, 43). Excess iron has been shown to increase the virulence of *pgm*-deficient *Y. pestis* strains in both mice and humans (44–47), well supporting this conclusion, although the *pgm* locus has additional functions beyond Ybt production (48, 49) In addition, iron chelation therapy only extended the survival period and slightly improved systemic infection, emphasizing that immune dysregulation driven by high iron and Ybt in ∆*fur*-HH mice is irreversible. Given those, we speculate that young adult HH mice used in our studies likely display dysregulated inflammatory responses to high Ybt-producing strains, amplifying disease severity while still retaining the capacity to control non- or low Ybt-producing strains. Therefore, host iron levels are critical determinants of infection progression, and genomic differences among *Yersinia* species are also important factors to consider.

Localized coagulation restricts bacterial dissemination (50, 51), while its dysregulated activation transforms into systemic immune thrombosis, a defining feature of sepsis pathogenesis (52, 53). Pharmacological inhibition of coagulation by rivaroxaban alleviated thrombosis but failed to improve survival. This may be due to rivaroxaban’s limited effect on key components of the inflammatory response in sepsis, such as endotoxin-induced cytokine production, leukocyte activation, and monocyte tissue factor expression (54). Additionally, coagulation may be a downstream consequence of uncontrolled activation of upstream immune mediators.

Transcriptomic and immunologic profiling revealed that ∆*fur*-induced systemic infection in HH mice was dominated by proinflammatory mediators, excessive neutrophil infiltration, neutrophil hyperactivation, and a sharp decline in CD4^+^ T cells. These are hallmarks of immune dysregulation that together shift the immune system toward a hyperinflammatory yet functionally compromised state (55). The surge in circulating pathogen- and damage-associated molecular patterns could fuel the increased neutrophil mobilization and recruitment. In parallel, sepsis causes a stochastic loss of CD4 T cells through apoptosis via multiple pathways, including the extrinsic (death receptor) pathway and the intrinsic (mitochondrial) pathway (56–58). It is well documented that iron overload alters death receptors and mitochondria (59). Therefore, we reason that *Yersinia* or other bacterial infections in iron overload hosts exacerbate aberrant alterations, leading to detrimental outcomes. However, the exact reasons remain to be further investigated.

Among immune cells engaged in the host response to severe systemic infection or sepsis, neutrophils have long been recognized for their pathological contributions, exacerbating disease severity through mechanisms such as neutrophil extracellular trap (NET) formation (60, 61), proinflammatory cytokine secretion (62), and T cell inhibition (63). Notably, neutrophil depletion mitigated DIC and improved survival, underscoring neutrophils as critical drivers of disease exacerbation in ∆*fur*-infected HH mice. Specifically, we found that CD101^−^ neutrophils play a key role in severe systemic infection and sepsis in HH hosts. CD101 was identified as a marker of neutrophil maturation, distinguishing immature Ly6G^low/+^CXCR2^−^CD101^−^ from mature Ly6G^+^CXCR2^+^CD101^+^ neutrophils (15). Under steady-state conditions, CD101^−^ immature neutrophils are rare in circulation but expand in inflamed tissues and have been associated with other inflammatory pathologies, such as tumor progression (16) and ischemia (64). In sepsis, rapid neutrophil expansion depletes bone marrow reserves of mature neutrophils, prompting the release of immature neutrophils into circulation and promoting systemic spread (55, 65, 66). Consistent with the findings, ∆*fur* infection in HH mice triggered massive recruitment of immature CD101^−^ neutrophils to the liver, blood, and spleen, whereas naïve HH and B6 mice, as well as ∆*fur*-infected B6 mice, predominantly contained mature CD101^+^ neutrophils. Intriguingly, the predominance of mature CD101^+^ neutrophils in naïve HH mice, along with their comparable bactericidal capacity to that of naïve B6 mice, suggests that *Hfe* deficiency does not promote excessive neutrophil recruitment or expansion of the immature CD101^−^ subset, nor does it markedly affect neutrophil function in young adult mice under homeostatic conditions. The CD101^−^ neutrophils exhibited reduced bactericidal capacity but elevated proinflammatory cytokine production, mirroring the immunopathogenic neutrophil phenotypes seen in human sepsis and cancer (67). Notably, 90% of bone marrow neutrophils in ∆*fur*-infected HH mice were CD101^−^, indicating emergency granulopoiesis and impaired host defense (65).

Unlike infected B6 mice, HH mice failed to control ∆*fur* replication, resulting in excessive Ybt production. This promoted bacterial dissemination and enhanced TLR4-mediated type I IFN signaling, driving the expansion of immature CD101^−^ neutrophils. Inhibiting TLR4 or infection with LPS-detoxified ∆*fur* mutants reduced type 1 IFN levels, limited CD101^−^ neutrophil recruitment, and improved survival. Likewise, loss of Ybt, the iron-scavenging molecule, attenuated type I IFN responses and improved outcomes, highlighting the cooperative roles of Ybt and TLR4 signaling in disease progression. It is unclear whether Ybt can directly trigger type I IFN, but its effects on pyroptosis and mitochondrial dysfunction (39, 68) likely amplify IFN signaling. In addition, MLCK and IL-1β neutralization improved barrier integrity and survival of ∆*fur*-infected HH mice (12). We reason that type I IFN signaling is an upstream driver of MLCK and IL-1β overproduction. Blocking IFNAR signaling reduced MLCK expression, restored barrier integrity, limited bacteria dissemination, and prevented mobilization of pathogenic CD101^−^ neutrophils from the bone marrow while preserving the appropriate mobilization of CD101^+^ neutrophils, thereby enhancing bacterial clearance.

Altogether, our findings demonstrate that Ybt overproduction enhances bacterial virulence, an effect that is further amplified in iron-overloaded hosts. Uncontrolled Δ*fur* infection in HH mice triggers emergency granulopoiesis followed by sepsis, characterized by a marked expansion of CD101^−^ neutrophils across multiple organs. Excessive type I IFNs, driven by TLR4 activation and Ybt from the Δ*fur* strain, fuel the recruitment of pathogenic CD101^−^ neutrophils, which act as key mediators of this detrimental infection.

### Limitations of this study

Our study has limitations. Without CD101 knockout mice, we used magnetic and FACS sorting to isolate CD101^+^ and CD101^−^ neutrophils with ~90% purity, though minor contamination may have affected responses to ∆*fur*. The short neutrophil lifespan limited *ex vivo* assays to 4–12 h, and ∆*fur*-induced neutrophil necrosis complicated distinguishing active cytokine secretion from passive leakage. In addition, we did not examine the phenotypes of CD101^−^ neutrophils in other iron-overloaded models infected with either high- or non-Ybt-producing strains. We will investigate these in future studies.

## MATERIALS AND METHODS

### Bacterial strains and culture conditions

*Y. pseudotuberculosis* ∆*fur*, ∆*fur* ∆*irp2*, or ∆*fur* ∆*lpxP* ∆*lpxL* mutants were constructed and cultured as mentioned in methods section earlier (12). *Y. pseudotuberculosis* and *Y. enterocolitica* strains used in this study were routinely grown in Luria-Bertani ( LB) broth or on LB agar plates at 28°C.

### Animal experiments

Mice were deprived of food and water for 6 h and then administered 200 µL of PBS containing 5 × 10^7^ colony-forming units (CFUs) of ∆*fur* as indicated by oral gavage. The mobility and mortality of infected animals were monitored for 15 days and bacterial burden in different tissues was determined. To determine the bacterial burden after infection, animals were euthanized with CO_2_ on the indicated days. Liver and spleen (approx. 1 g) were collected and homogenized in ice-cold PBS using a bullet blender (Bullet Blender Blue; NY, USA) at power 7 for 2 min. Blood (~100 µL) was collected in EDTA-containing tubes by submandibular puncture. Serial dilutions of the blood or tissue homogenates were plated on LB agar plates with kanamycin, and each count was confirmed from duplicate plates with three dilutions to determine the bacterial titer per gram of tissue or milliliters of blood. Intestinal tight junction disruption and MLCK expression in liver and intestine were evaluated by western blot analysis as mentioned in methods section earlier (12).

### Liver whole transcriptome analysis

Uninfected or Δ*fur-*infected HH mice (*n* = 3 each group) were euthanized at 6 dpi; their liver was perfused with PBS, harvested, and stored in RNAlater at −80°C. Total RNA was extracted from frozen intestine sections using an RNAeasy mini kit (Qiagen, USA) following the manufacturer’s instructions. To remove trace DNA, a column DNase treatment was performed using RNase-free DNase according to the manufacturer’s instructions. The RNA-seq libraries and data were generated at the University at Albany’s Center for Functional Genomics shared resource facility (RRID: SCR_018262). The quality of total RNA was evaluated using an Agilent 2100 Bioanalyzer (Agilent Technologies, USA). A complementary DNA library was prepared using an NEB Ultra II Directional Kit, and sequencing was performed on the Nextseq500 system according to Illumina’s standard protocol (https://en.novogene.com/). After sequencing, individual base calls were demultiplexed and assigned to fastq files using Illumina’s bcl2fastq program. Raw fastq files were then assessed for quality using FASTQC. Following evaluation of quality and control, RNA-Seq libraries were aligned to the GRCm38/mm10 mouse reference genome, and gene counts were generated using STAR. The RNA-seq data were uploaded to the GEO database and were available under accession number GSE300863. Analysis of differential gene expression was performed using the DESeq2 package in R. Significant DEGs were identified as ±2 log_10_ fold change. Heatmaps were generated using heatmap, and dot plots were generated using ggplot26.

### Gene ontology and KEGG enrichment analyses

Significant DEGs were subjected to GO enrichment analysis by the GOseq R Package to correct for the gene length bias. GO analysis was performed using the GeneOntology online tool powered by Panther (http://geneontology.org/). The top GO categories were identified according to the P-value score. Pathway analysis of the significant DEGs was performed using the KEGG database (https://www.kegg.jp/kegg/). Statistical enrichment of the DEGs in KEGG pathways was tested using KOBAS.

### Histopathology analysis

Liver and spleen tissues were perfused with PBS, excised aseptically from euthanized mice, and fixed in 10% buffered formalin for 48 h. The tissue sections were embedded in paraffin, sectioned at 5 μm thickness, placed on ultraclean glass slides, and stained with H&E. Images were captured using a NanoZoomer 2.0 RS Hamamatsu slide scanner. For immunofluorescence staining, tissue sections were subjected to antigen retrieval in Tris-EDTA buffer (pH 9.0), washed with 1× PBS, blocked in 5% bovine serum albumin (BSA), and incubated with the appropriate antibodies in 0.5% BSA. After incubation with the appropriate antibodies, the tissue sections were washed and probed with a fluorescent dye-conjugated secondary antibody in 0.5% BSA and incubated. Subsequently, the tissue sections were washed and mounted under a coverslip using Flourshield with DAPI and examined under an Echo Revolve R4 fluorescence microscope (Echo, USA).

### Tail bleeding assay

Mice were anesthetized with a concoction of 25% ketamine and 12.5% xylazine in sterile PBS and placed in an upright position. A 10 mm segment at the distal end of the tail was amputated with a sterile scalpel and immersed immediately in a 50-mL Falcon tube containing pre-warmed PBS at 37°C. Tail bleeding was monitored for 20 min, even if the bleeding ceased earlier, to detect re-bleeding. In each mouse, the time taken for bleeding to cease was determined with a stop clock. The experiment was terminated at the end of 20 min to avoid lethality during the experiment, and the mouse tail was treated with mupirocin to prevent infection.

### Platelet count and analysis

Blood was collected in EDTA-containing tubes by submandibular puncture with a sterile 4 mm scalpel. The platelet numbers were determined by performing a complete blood count on a Hema Vet 950 analyzer.

Blood was collected from mice as mentioned above; platelets were purified from the plasma and resuspended in Tyrode’s buffer (0.34 mM Na_2_HPO_4_, 134 mM NaCl, 2.9 mM KCl, 12 mM NaHCO_3_, 5mM glucose, 20 mM HEPES, pH 7.0, and 0.35% bovine serum albumin). Purified platelets were stained with CD42b, anti-CD62P mouse antibodies, and subjected to flow cytometry. For the platelet activation assay, thrombin (0.05 U/mL, Sigma) was added together with antibodies and incubated at room temperature for 15 min. The reaction was stopped by adding PBS, and the activated platelets were analyzed by flow cytometry within 30 min.

### ELISA assays

Mice were bled by submandibular puncture; serum was extracted by incubating blood samples at 37°C for 20 min, followed by centrifugation at 8,000 rpm for 10 min. Serum was stored at −20°C until further use. Serum samples were used to detect ALT, AST, BUN, creatine, F3, fibrin, and D-dimer via ELISA kits (MyBiosource, USA) as per the manufacturer’s instructions.

### Preparation of single-cell suspensions

Spleen or liver tissues were excised from euthanized mice and collected in ice-cold PBS. Tissue sections were homogenized in PBS + 0.5% BSA (FACS buffer) using a 40 µm strainer. Bone marrow cells were obtained by flushing the femur and tibia with PBS + 0.5% BSA buffer, filtered through 18 g needles, and passed through a 40 µm strainer.

### Flow cytometry analysis

A single-cell suspension was prepared from the liver, spleen, blood, and bone marrow as described above and subjected to erythrocyte (RBC) lysis using ACK buffer. Cells were resuspended in FACS buffer for all subsequent procedures. To minimize non-specific antibody interactions, Fc-receptors on cells were masked with Fc block CD16/32. The cells were surface stained with the fluorescently labeled antibodies (Table S1) in FACS buffer at 4°C for 30 min in the dark. Subsequently, cells were washed with FACS buffer and fixed with Fixation buffer as per the manufacturer’s instructions. Events were acquired on the BD FACSymphony flow cytometer with FACSDiva software and were analyzed using FlowJo v.10. Dead cells were excluded by staining with eFluor 780-conjugated viability dye, followed by additional gating to assess various populations of cells. The total live neutrophil counts were normalized to the tissue weight and plotted as the number of live neutrophils per gram of tissue.

### Isolation of total neutrophils and CD101^+^ and CD101^−^ neutrophils

Single-cell suspensions were made as mentioned in the paragraph above, subjected to RBC lysis with ACK buffer, and then resuspended in 1× MojoSort buffer. Neutrophils were isolated (~95% purity) by negative selection using the MojoSort Mouse Neutrophil Isolation kit following the manufacturer’s instructions. Isolated neutrophils were further stained with CD101-PE antibody in FACS buffer at 4°C for 30 min in the dark, washed, and resuspended in FACS buffer. LiveCD101^+^ and LiveCD101^−^ neutrophils were sorted using the FACS Aria cytometer with FACSDiva software. Sorted cells were collected in FACS buffer at 4°C for subsequent assays.

### Quantification of cytokines/chemokines

Liver of ∆*fur*-infected B6 and HH mice was perfused with PBS to prevent RBC contamination, excised, and homogenized in ice-cold PBS with protease inhibitor at 4°C. The liver homogenates were filtered, and the total protein was determined using BCA assay (Pierce, USA). The cytokine/chemokine profiles as indicated were determined using the Bio-Plex Pro Mouse Cytokine 23 Plex assay (BioRad) using the manufacturer’s instructions. For culture supernatants, purified CD101^+^ and CD101^−^ neutrophils (4 × 10^6^ cells/well) were infected with ∆*fur ex vivo* for 2 h. Subsequently, supernatants were collected, passed through 0.22 µM filters, and subjected to cytokine analysis as mentioned above.

### Neutralization assays

HH mice were administered intraperitoneally with mouse anti-Ly6G (200 µg/mouse, clone 1A8) or anti-IFNAR (2.5 mg/kg, clone MAR1-5A3), neutralizing antibody in 200 µL sterile 1× PBS on 0, 2, 4, and 6 dpi. Control mice received rat IgG2a isotype control antibodies (clone 2A3).

### TLR4 inhibition

HH mice were administered with TLR4 antagonist Tak-242 (3 mg/kg, Cayman) in 200 µL vehicle solution, twice weekly by i.p. Control mice received 200 µL vehicle solution (10% DMSO, 10% PBS + 1% Tween 80) on similar dpi.

### Bactericidal assays

Neutrophils derived from blood or bone marrow (4 × 10⁶ cells/well) were infected with the ∆*fur* strain at a multiplicity of infection of 5 and incubated at 37°C in 5% CO_2_. For the phagocytosis assay, after 2 h of incubation, culture supernatants containing extracellular, non-phagocytosed bacteria were discarded. Cells were washed with PBS and centrifuged at 300 × *g* to remove any remaining extracellular bacteria. The resulting cell pellet was lysed in PBS containing 0.1% Triton X-100 and centrifuged at 4,000 × *g* to collect the phagocytosed (intracellular) bacterial pellet. This pellet was resuspended in PBS and plated on LB agar to determine the colony-forming units representing total phagocytosed bacteria. To assess bactericidal activity, ∆*fur*-infected neutrophils were incubated in DMEM medium containing gentamicin for an additional 2 h (total incubation time: 4 h). The infected cells were then lysed and processed as described above for CFU enumeration. Bactericidal activity was calculated by comparing the CFU count obtained after the 4-h incubation (bactericidal assay) to the CFU count from the 2-h phagocytosis assay.

### Statistical analysis

Statistical analyses of comparisons of data among groups were performed with one-way ANOVA/univariate or two-way ANOVA with the Tukey post-hoc test. The log-rank (Mantel-Cox) test was used for survival analysis. All data were analyzed using GraphPad PRISM 8.0 software. Data are presented as the mean ± standard deviation (ns, no significance; *P < 0.05; **P < 0.01; ***P < 0.001; ****P < 0.0001).

## SUPPLEMENTAL MATERIAL

Data set S1 iai.00691-25-s0001.xlsx DeSeq analysis for liver whole transcriptome analysis.

Data set S2 iai.00691-25-s0002.xlsx Whole blood CBC of infected HH mice treated with α-IFNAR or IgG antibodies.

Supplemental material iai.00691-25-s0003.docx Supplemental text, table, and figures.

ASM does not own the copyrights to Supplemental Material that may be linked to, or accessed through, an article. The authors have granted ASM a non-exclusive, world-wide license to publish the Supplemental Material files. Please contact the corresponding author directly for reuse.
