# Supplementary material for: Expansion of CD101⁻ neutrophils drives susceptibility to hyperyersiniabactin-producing Yersinia infection in hereditary hemochromatotic hosts
Source: Infect Immun. 2026 Jun 12;94(7):e00691-25. doi: 10.1128/iai.00691-25 (PMC13367047; doi:10.1128/iai.00691-25)
Supplement: Supplemental material — Supplemental text, table, and figures. [file iai.00691-25-s0003.docx]

**Supplemental Information**

**Expansion of CD101⁻ neutrophils drives susceptibility to hyper-yersiniabactin-producing *Yersinia* infection in hereditary hemochromatotic hosts**

Shreya Das^1^, Saugata Majumder^1^, Mohd Saqib^2^, McKenzie Van der Veer ^1^, Bossardi Ramos, Ramon^2^, Akaki Tsilosani^3^, Wenzheng Zhang^3^, Qi Yang ^4^, Sridar V Chittur^5^ and Wei Sun^1#^

1. Department of Immunology and Microbial Disease, Albany Medical College, Albany, NY, 12208, USA. 2. Department of Molecular and Cellular Physiology, Albany Medical College, Albany, NY, 12208, USA. 3. Department of Regenerative & Cancer Cell Biology, Albany Medical College, Albany, NY, 12208, USA. 4. Rutgers Institute for Translational Medicine and Science, Child Health Institute of New Jersey, The State University of New Jersey, New Brunswick, NJ 08901, USA. 5. Center for Functional Genomics, University at Albany-State University of New York, Rensselaer, NY 12144.

**Running Title:**

Pathogenic role of immature CD101⁻ neutrophils in hemochromatotic mice during infection

# Corresponding author: Department of Immunology and Microbial Disease, Albany Medical College, Albany, NY, 12208. Email: sunw@amc.edu.

Conflict of interest: All other authors declare that they have no conflicts of interest.

**Supplementary Materials and Methods**

**Table S1: Key Resources Table**

| **Reagent or Resource** | **Source** | **Identifier** |
| --- | --- | --- |
| **Chemicals, inhibitors and Recombinant proteins** | | |
| Flourshield^TM^ with DAPI | Sigma | Cat# F6057 |
| RNase-free DNase | Qiagen | Cat# 79254 |
| Protease inhibitor cocktail | Sigma | Cat# 04693132001 |
| ACK lysis buffer | Lonza | Cat# |
| Fixation buffer | Biolegend, | Cat# 420801 |
| RNAlater | Thermo Scientific, | Cat# AM7024 |
| DMEM | Corning, | Cat# 10-013-CV |
| Liberase TL | Sigma, | Cat# 05401020001 |
| Recombinant murine IFNα | Biolegend | Cat#752806 |
| Lipopolysaccharide (LPS) | InvivoGen | Cat# Tlrs-eblps |
| Fixable viability dye; eFlour 780 conjugated | eBioscience | Cat# 65-0864-14 |
| PBS | Sigma, | Cat# P5493 |
| Mojosort buffer | Biolegend | Cat#480017 |
| Thrombin | Sigma, | Cat#9002-04-4 |
| DMSO | Sigma, | Cat#D2650 |
| Triton X-100 | Sigma, | Cat# 93443 |
| Rivaroxaban | Cayman | Cat# 16043 |
| TAK-242 | Cayman | Cat# 13871 |
| Recombinant mouse GCSF | Peprotech | Cat# 250-05 |
| **Commercial kits and assays** | | |
| Mojo sort Neutrophil Isolation kit | Biolegend, | Cat# 480058 |
| Bio-Plex Pro mouse cytokine Grp; 23 plex | BioRad | Cat# M6000RDPD |
| Pierce BCA kit | ThermoFisher | Cat#23225 |
| Mouse Coagulation Factor III (F3) ELISA Kit | MyBiosource | Cat# MBS268199 |
| Mouse Alanine Aminotransferase (ALT) ELISA Kit | MyBiosource | Cat# MBS264717 |
| Mouse AST (Aspartate Aminotransferase) ELISA Kit | MyBiosource | Cat# MBS2510226 |
| Mouse Blood Urea Nitrogen (BUN) ELISA Kit | MyBiosource | Cat# MBS2611085 |
| Mouse Creatinine ELISA Kit | MyBiosource | Cat# MBS3805641 |
| Mouse CD142(F3) ELISA Kit | MyBiosource | Cat# MBS8244605 |
| Mouse fibrin ELISA Kit | MyBiosource | Cat# MBS706338 |
| Mouse D-Dimer (D2D) ELISA Kit | MyBiosource | Cat# MBS269348 |
| Mouse IFN alpha ELISA Kit | ThermoFisher | Cat# BMS6027 |
| RNAeasy mini kit | Qiagen, | Cat#74104 |
| Iron Assay kit | Sigma | Cat#MAK472 |
| **Antibodies** | | |
| Rat IgG2a isotype control | BioXcell | clone 2A3 |
| Anti-mouse Ly6G | BioXcell | clone 1A8 |
| Anti-mouse IFNAR | BioXcell | clone MAR1-5A3 |
| Anti- mouse GCSF | ThermoFisher Scientific | Cat#MA523758 |
| Fc block CD16/32 | Biolegend | Cat#156604 |
| CD45 | Biolegend | clone 145 |
| CD11b | Biolegend | clone M170 |
| Ly6G | Biolegend | clone 1A8 |
| Ly6C | Biolegend | clone HK1.4 |
| CD64 | Biolegend | clone |
| CD3 | Biolegend | clone 17A2 |
| CD101 | ThermoFisher Scientific | Moushi101 |
| GR1 | Biolegend | clone RB6-8C5 |

**Methods:**

**Bacterial strains and culture conditions.** The *Y. pseudotuberculosis* ∆*fur* ∆*irp2*, or ∆*fur* ∆*lpxP* ∆*lpxL* mutants were constructed and cultured as mentioned earlier (1). The *Y. enterocolitica* (Ye) WA strain used in this study was routinely grown in LB broth or on LB agar plates at 28 °C.

**Animal experiments.** Mice were deprived of food and water for 6 h and then administered 200 µl of PBS containing 5 × 10^5^ CFU of Ye WA or 5 × 10^7^ CFU of ∆*fur* ∆*irp2*, or ∆*fur* ∆*lpxP* ∆*lpxL* as indicated by oral gavage (o.g.).

**Iron assay.** The iron contents in mouse tissues and serum were measured using an iron assay kit. Briefly, 10 mg of spleen or liver tissue collected from euthanized mice was homogenized in 0.2 ml iron assay buffer and centrifuged at 16,000 ×g for 10 min at 4 °C to remove insoluble tissues, and the supernatant was used in the assay according to the manufacturer’s instructions. Serum samples were directly used for the assay.

**Iron inhibition therapy.** Iron-reduced HH mice were generated by intraperitoneal administration of 3 mg/dose deferoxamine (DFO) diluted in 1× PBS daily for 5 days before infection (2). Control mice received 200 µl vehicle solution (sterile 1× PBS).

**Rivaroxaban therapy.** HH mice were administered Rivaroxaban (100 µg/mouse, Cayman), a specific Factor Xa inhibitor, in 200 µl vehicle solution through oral gavage (o.g.) on 0, 2, 4, and 6 dpi. Control mice received 200 µl vehicle solution (10% DMSO, 10% PBS+1% Tween 80) at the same time points.

**GCSF neutralization assay.** HH mice were administered intraperitoneally with anti-GCSF (2.5 mg/kg) on 0, 2, 4, and 6 dpi. Control mice received rat IgG isotype antibodies (clone 2A3).

**Recombinant mouse G-CSF (rm-GCSF) administration.** HH mice infected with Δ*fur* were administered recombinant mouse G-CSF (r-GCSF, 1μg/mouse) intraperitoneally at 0, 2, and 4 dpi. Mice were euthanized on 6 dpi for analysis.

**LPS administration.** HH mice infected with the Δ*fur* Δ*lpxP*Δ*lpxL* mutant were administered 20 µg of *E. coli* LPS O111:B4 (Sigma) in 200 µL PBS per mouse via oral gavage at 0 and 7 dpi.

**Supplemental Figures and Figure Legends:**


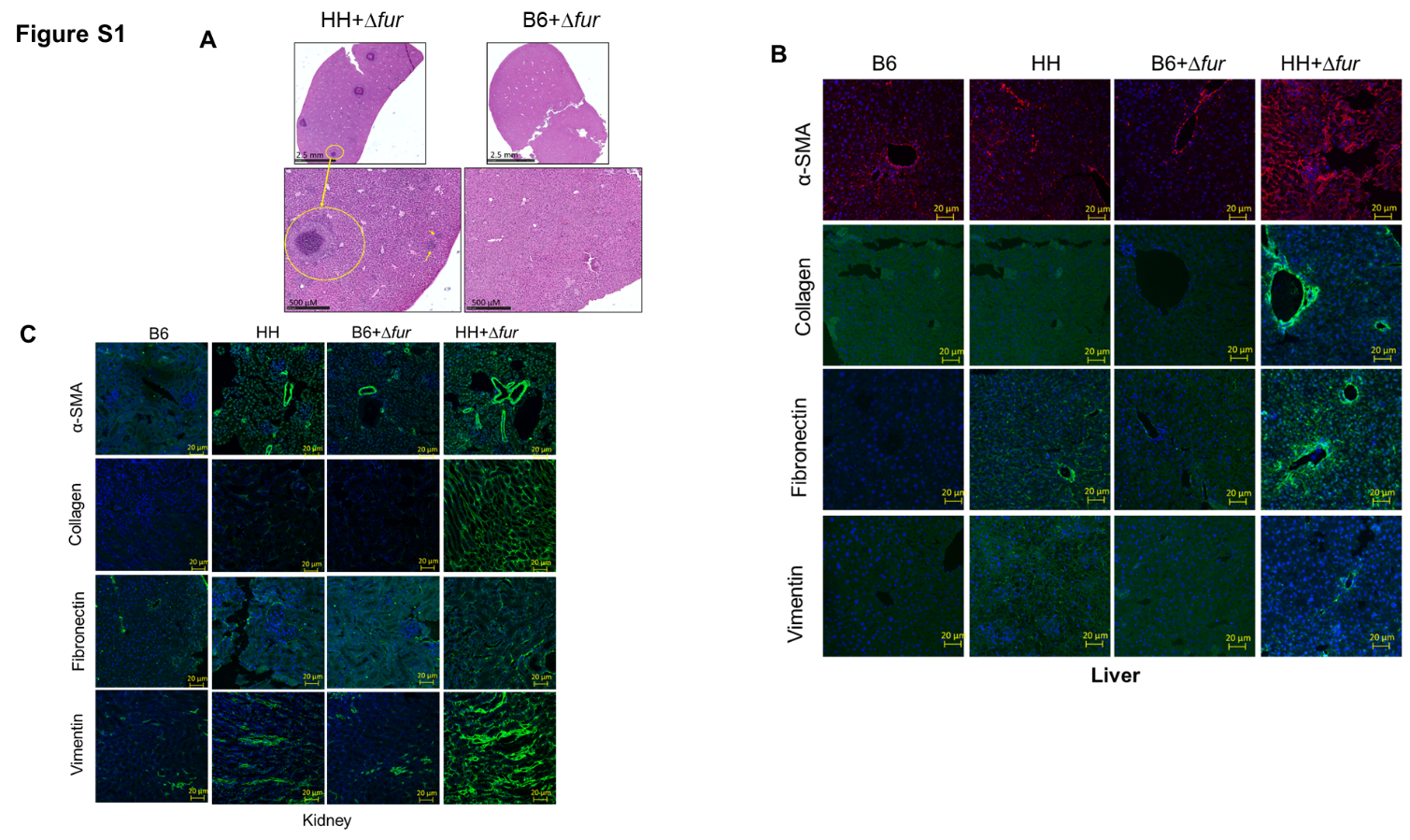


**Figure S1. Δ*fur* infection promotes systemic infection and DIC in HH mice.** (A-C) Liver histopathology from ∆*fur*-infected B6 and HH mice (n=3/group) at 6 dpi (A) Representative H&E staining of liver sections (B) Representative immunofluorescence staining of liver and (C) kidney sections.

**
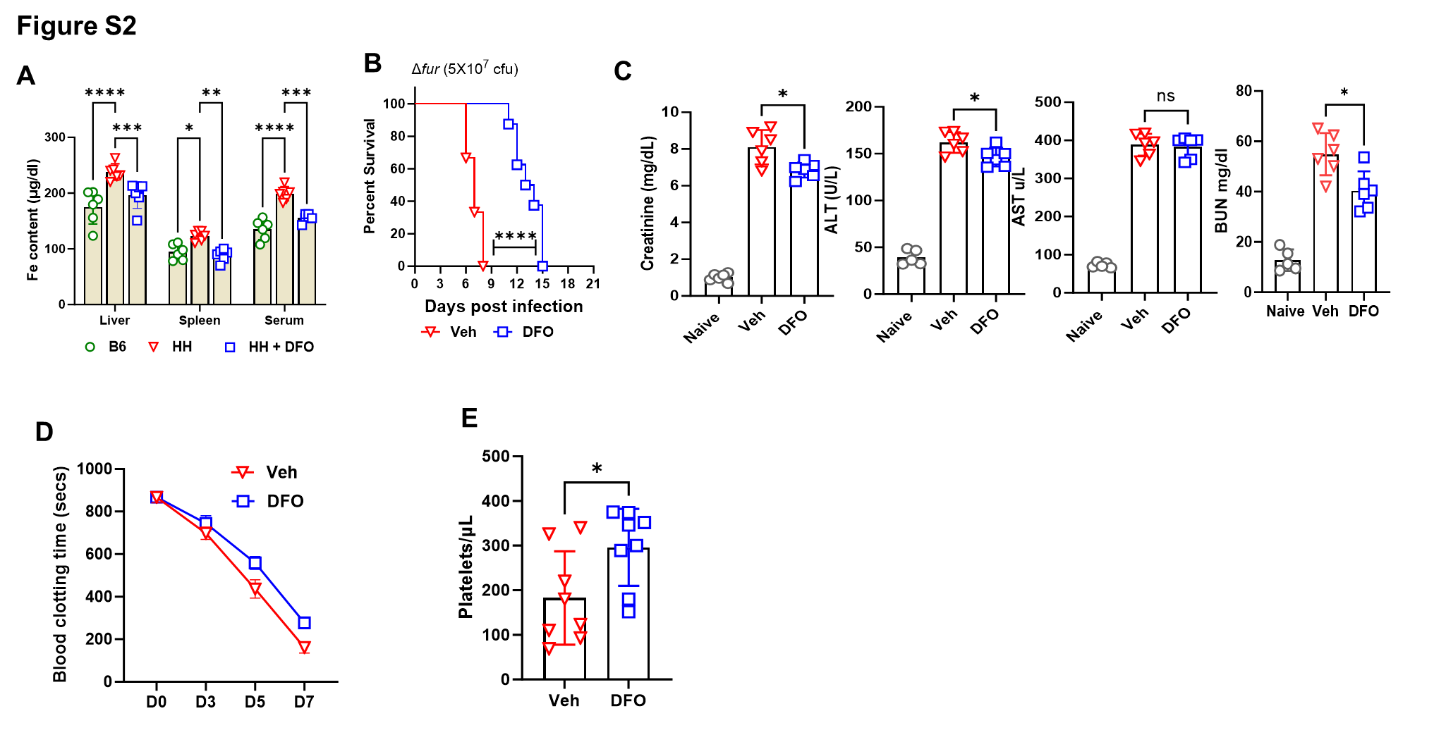
**

**Figure S2. Iron excess in the HH host promotes *fur*-induced hypervirulence and DIC.**  Inhibition of excess iron in HH mice by administering DFO (3 mg/dose in 200 µl sterile 1X PBS), intraperitoneally daily from -5 to 0 dpi. Control mice received 200 µl vehicle solution (sterile 1X PBS). (A) Systemic iron (Fe) content in naïve HH mice treated with DFO or vehicle control. Naïve B6 mice were used as controls for basal systemic iron levels. (B) Survival of ∆*fur*-infected HH mice treated with DFO or vehicle control. (C) Serum creatinine, ALT, AST, and BUN levels of ∆*fur* infected HH treated with DFO or vehicle control at 6 dpi. Serum samples from naïve B6 mice served as uninfected controls. (D) Time to cease bleeding in response to tail injury from ∆*fur-*infected HH mice treated with DFO or vehicle control at 6 dpi (n=6/group). (H) Platelet counts in the same mice at 6 dpi. For all panels, unless otherwise mentioned, each symbol represents data from an individual mouse. Statistical analyses of comparisons of data among groups were performed with unpaired t-test using a parametric test (C) or two-way ANOVA with the Tukey post-hoc test (A and D). The log-rank (Mantel-Cox) test was used for survival analysis (B). Data are presented as the mean ± standard deviation (ns, no significance; * *P*< 0.05; ** *P*< 0.01; *** *P*< 0.001; **** *P*<0.0001).

**
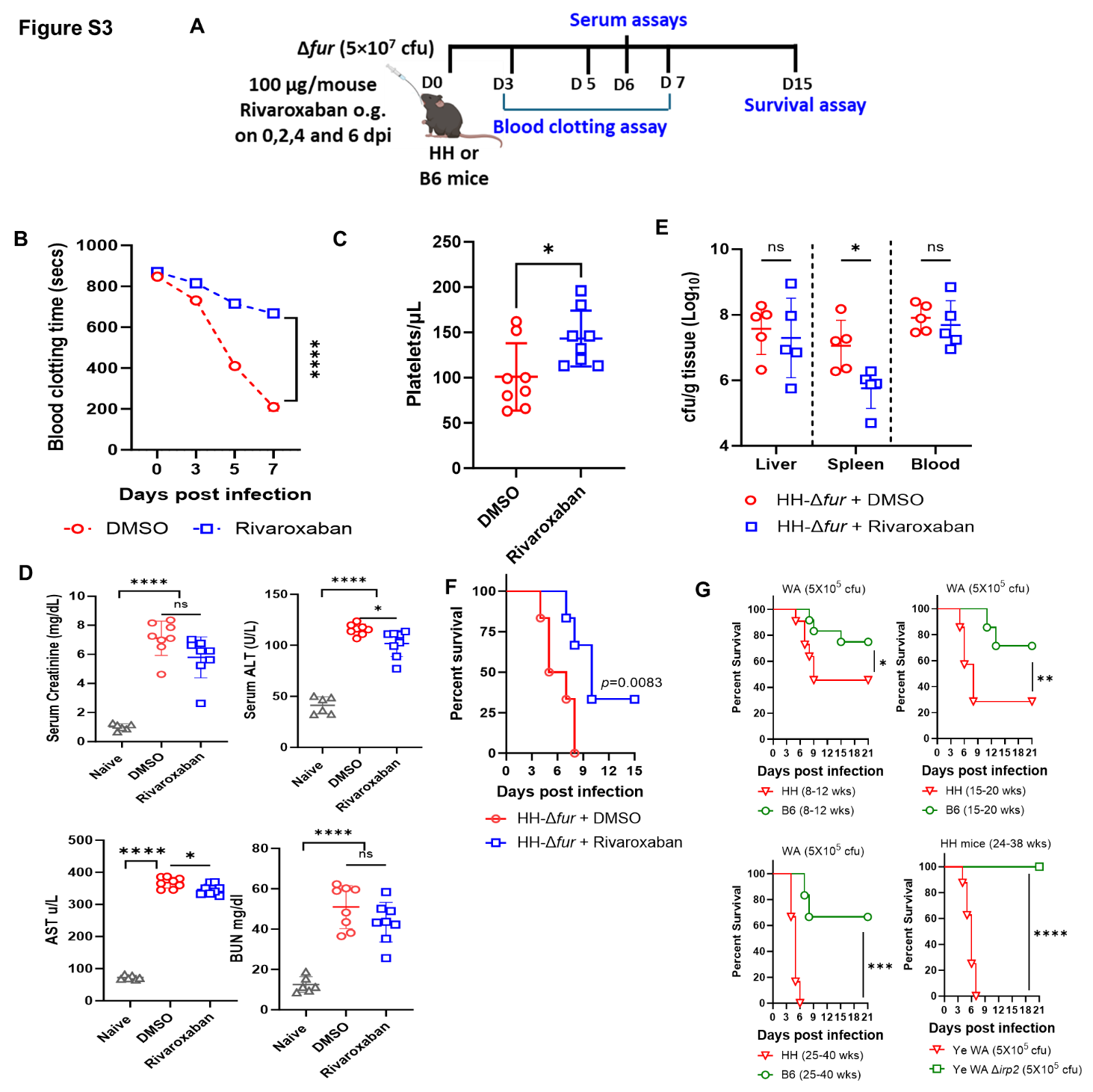
**

**Figure S3. Assessment of coagulation inhibition during Δ*fur*-induced sepsis in HH mice.** (A) Schematic illustrating inhibition of coagulation by administering Rivaroxaban in 200 µl vehicle solution through oral gavage (o.g.) in ∆*fur*-infected HH mice. Control mice received 200 µl vehicle solution (10% DMSO, 10% PBS + 1% Tween 80). (B) Coagulation assay. Time to cease bleeding in response to tail injury from ∆*fur*-infected B6 and HH mice (n=5). (C) Platelet counts at 6 dpi in ∆*fur*-infected HH mice treated with Rivaroxaban or vehicle control. (D) Serum ALT, AST, creatinine, and BUN levels in ∆*fur*-infected HH and B6 mice at 6 dpi. (E) Bacterial burden at 6 dpi in ∆*fur* infected HH mice treated with Rivaroxaban or vehicle control. (F) Survival of Δ*fur*-infected mice treated with Rivaroxaban or vehicle (n=6/group). (G) Survival of B6 and HH mice aged 8-12 weeks (n=12/group), 15-20 weeks (n=7/group), 25-40 weeks (n=6/group), infected with Ye WA and 24-28 week HH mice infected with Ye WA or Ye WA ∆*irp2*. For all panels, unless otherwise noted, each symbol represents data from an individual mouse. Statistical analyses of comparisons of data among groups were performed with an unpaired t-test using a parametric test (C) one-way ANOVA univariate (D) or two-way ANOVA with the Tukey post-hoc test (B and E). The log-rank (Mantel-Cox) test was used for survival analysis (F and G). Data are presented as the mean ± standard deviation (ns, no significance; * *P*< 0.05; ** *P*< 0.01; *** *P*< 0.001; **** *P*<0.0001).


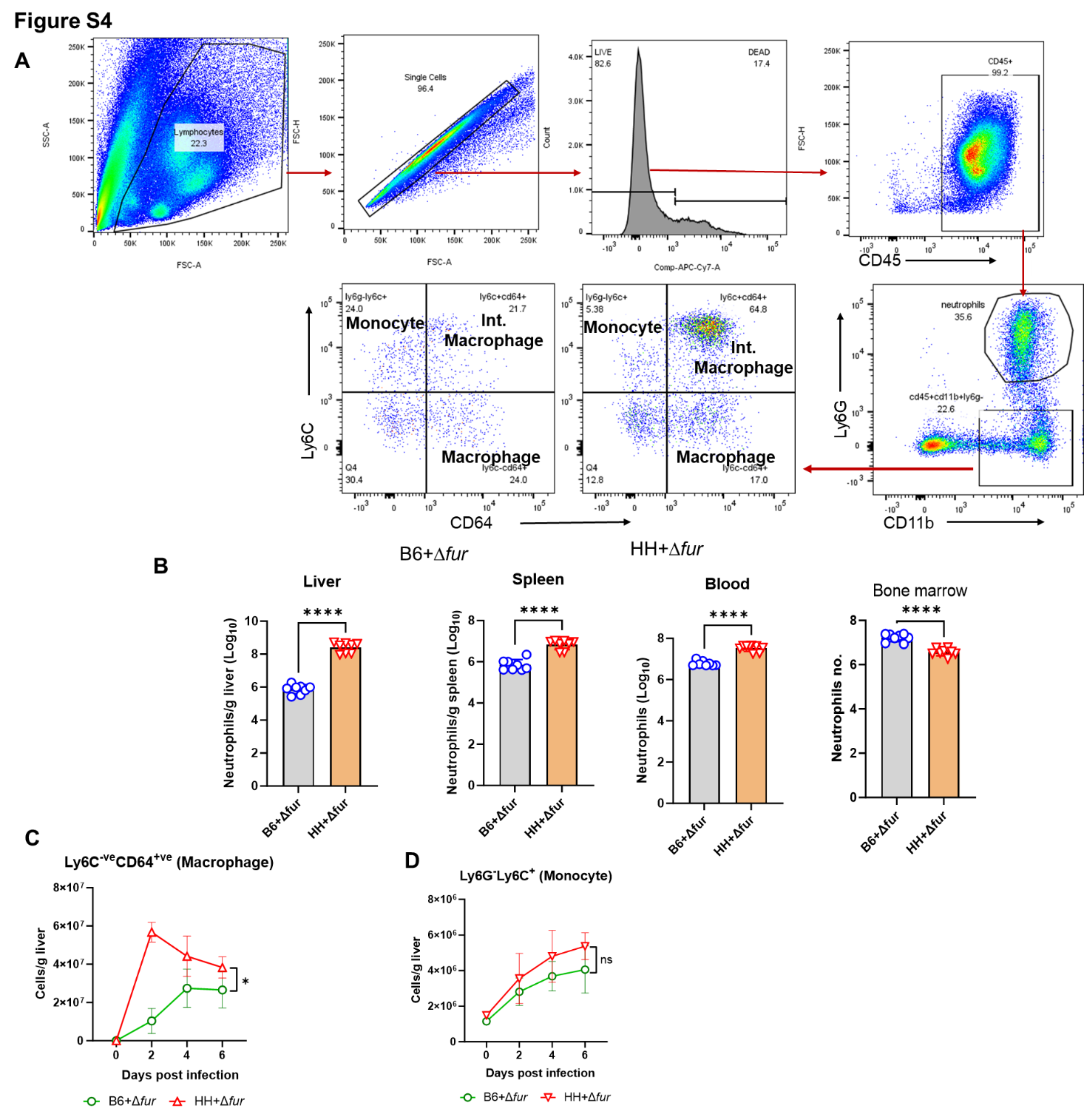


**Figure S4. Sepsis in Δ*fur*-infected HH mice correlates with high neutrophil influx**. (A) Gating strategy of live CD45^+^ cells to identify indicated populations. (B) Flow cytometric quantification of neutrophils in Δ*fur*-infected HH and B6 mice at 6 dpi. (C–D) Flow cytometric quantification of (C) macrophages and (D)monocytes in Δ*fur* infected HH and B6 mice at 0, 2, 4, and 6 dpi (n = 3/group). For all panels, unless otherwise noted, each symbol represents data from an individual mouse. Statistical analyses of comparisons of data among groups were performed with an unpaired t-test using a parametric test (B) or two-way ANOVA with the Tukey post-hoc test (C and D). Data are presented as the mean ± standard deviation (ns, no significance; * *P*< 0.05; ** *P*< 0.01; *** *P*< 0.001; **** *P*<0.0001).


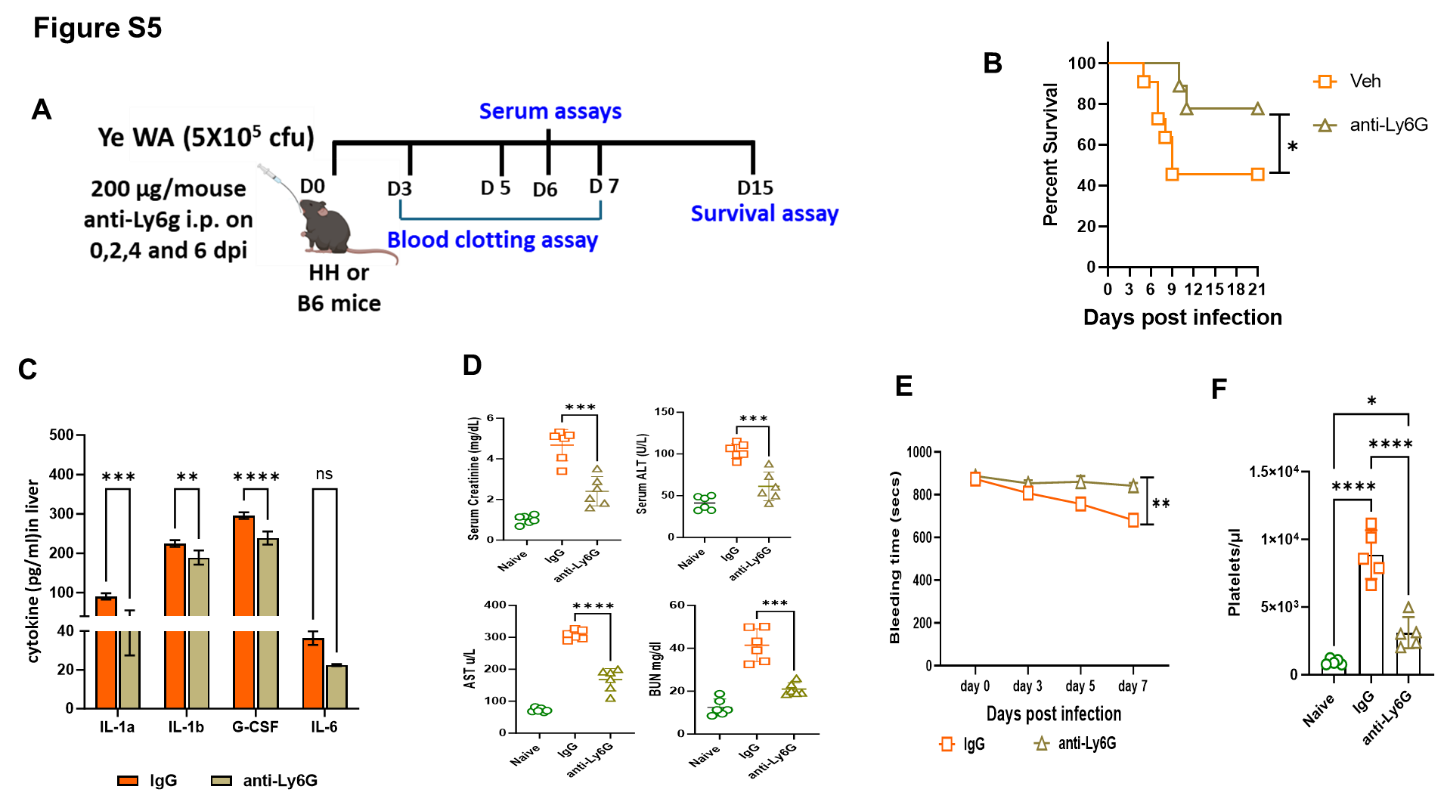


**Figure S5: Neutrophils play a critical role in exaggerating Ye WA infection in HH mice.** (A) Schematic illustrating neutrophil depletion in Ye WA–infected HH mice. (B) Survival of Ye WA–infected HH mice treated with mouse anti-Ly6G antibodies or rat IgG2a isotype control (n = 11 per group). (C) Cytokine levels in liver homogenates at 6 dpi from Ye WA infected HH mice treated with anti-Ly6G or isotype control IgG (n = 3/group). (D) Serum levels of creatinine, ALT, AST, and BUN were measured by ELISA. (E) Time to cessation of bleeding. (F) Platelet counts at 6 dpi in Ye WA infected HH mice treated with anti-Ly6G or IgG. For all panels, unless otherwise mentioned, each symbol represents data from an individual mouse. Statistical analyses of comparisons of data among groups were performed with an unpaired t-test using a parametric test (D), one-way ANOVA/univariate (F) or two-way ANOVA with the Tukey post-hoc test (C and E). The log-rank (Mantel-Cox) test was used for survival analysis (B). Data are presented as the mean ± standard deviation (ns, no significance; * *P*< 0.05; ** *P*< 0.01; *** *P*< 0.001; **** *P*<0.0001).


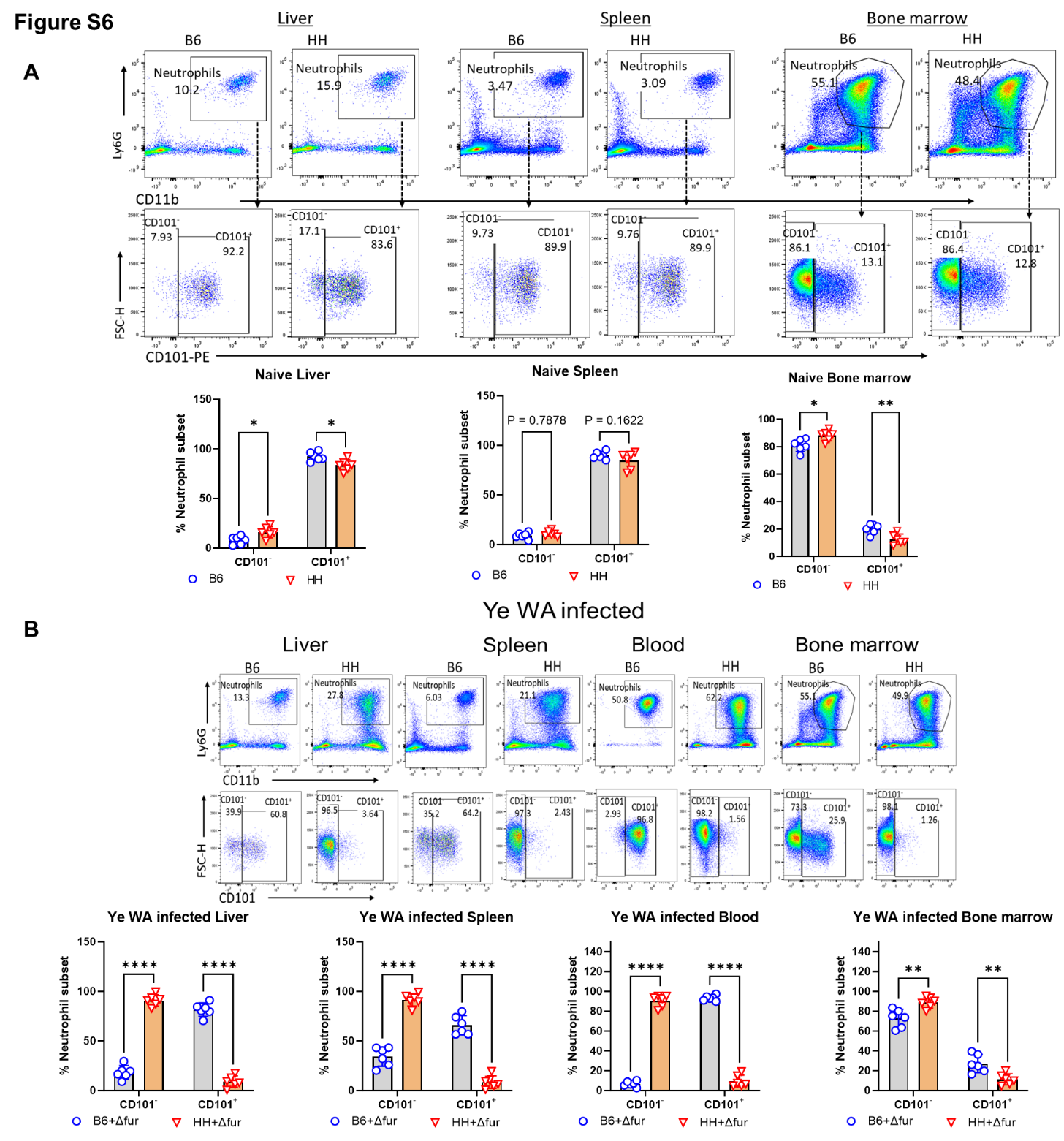


**Figure S6. CD101^+^ and CD101^-^ neutrophil subsets in HH and B6 mice.** (A, B) Representative fluorescence-activated cell sorting (FACS) plots and quantitative plots showing CD101 expression on neutrophils in various organs of (A) naive B6 and HH mice, and (B) Ye WA infected HH mice at 6 dpi. For all panels, unless otherwise mentioned, each symbol represents data from an individual mouse. Statistical analyses of comparisons of data among groups were performed with two-way ANOVA with the Tukey post-hoc test (A and B). Data are presented as the mean ± standard deviation (ns, no significance; * *P*< 0.05; ** *P*< 0.01; *** *P*< 0.001; **** *P*<0.0001).


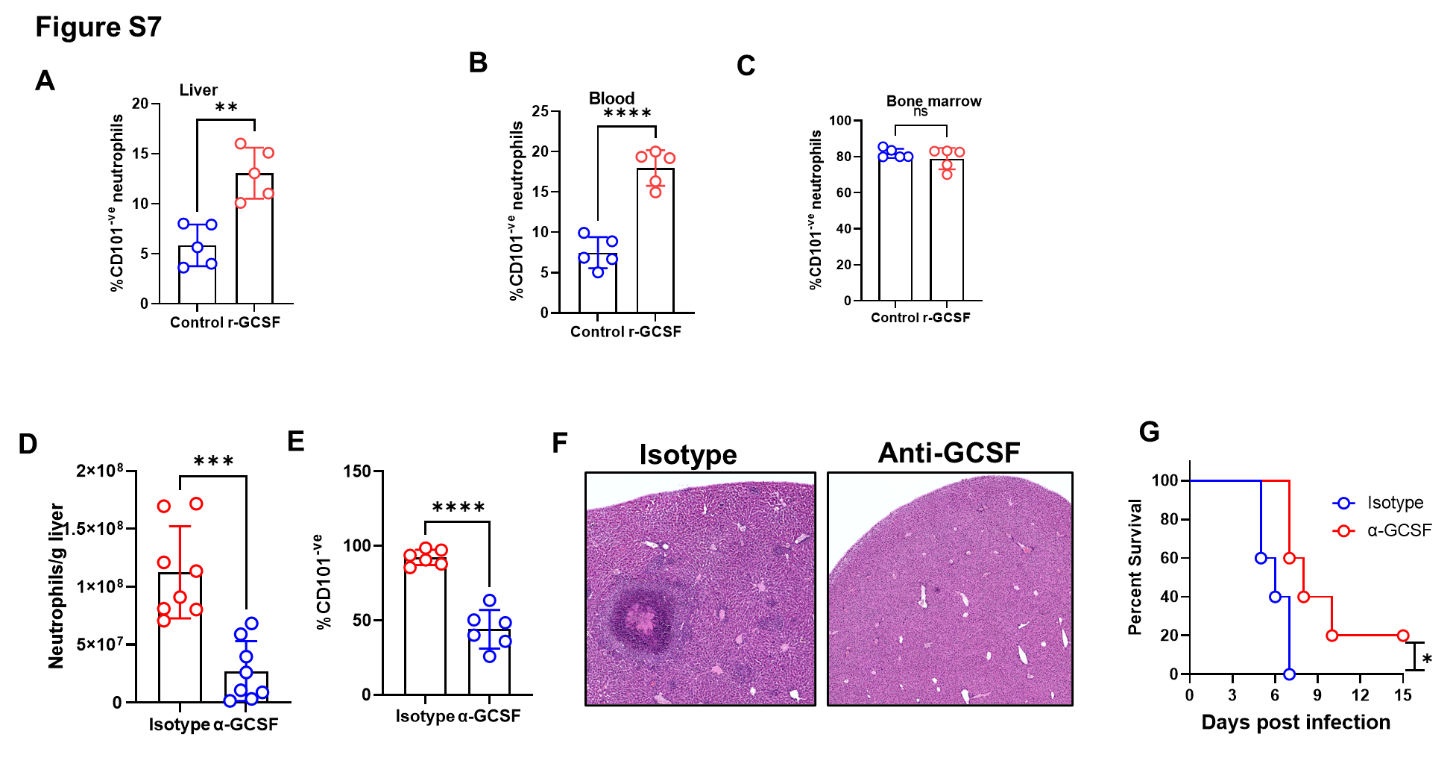


**Figure S7.** **G-CSF regulates migration of CD101^-^ neutrophils.** (A–C) Proportion of CD101⁻ neutrophils in the indicated organs of recombinant G-CSF or vehicle-treated mice at 6 dpi. (D-G) Schematic representing inhibition of G-CSF signaling by administration of anti-G-CSF neutralizing antibodies to Δ*fur* infected HH mice was treated with mouse anti-G-CSF antibodies or isotype control (rat IgG2a). (D) Total neutrophil counts and (E) Proportion of CD101⁻ neutrophils in the liver of treated mice at 6 dpi. (F) Representative H&E-stained images of liver sections from the same mice. (G) Survival analysis of Δ*fur*-infected HH mice treated with anti-G-CSF neutralizing antibodies or isotype control. Statistical analyses of comparisons of data among groups were performed with an unpaired t-test using parametric test. The log-rank (Mantel-Cox) test was used for survival analysis. Data are presented as the mean ± standard deviation (ns, no significance; * *P*< 0.05; ** *P*< 0.01; *** *P*< 0.001; **** *P*<0.0001).


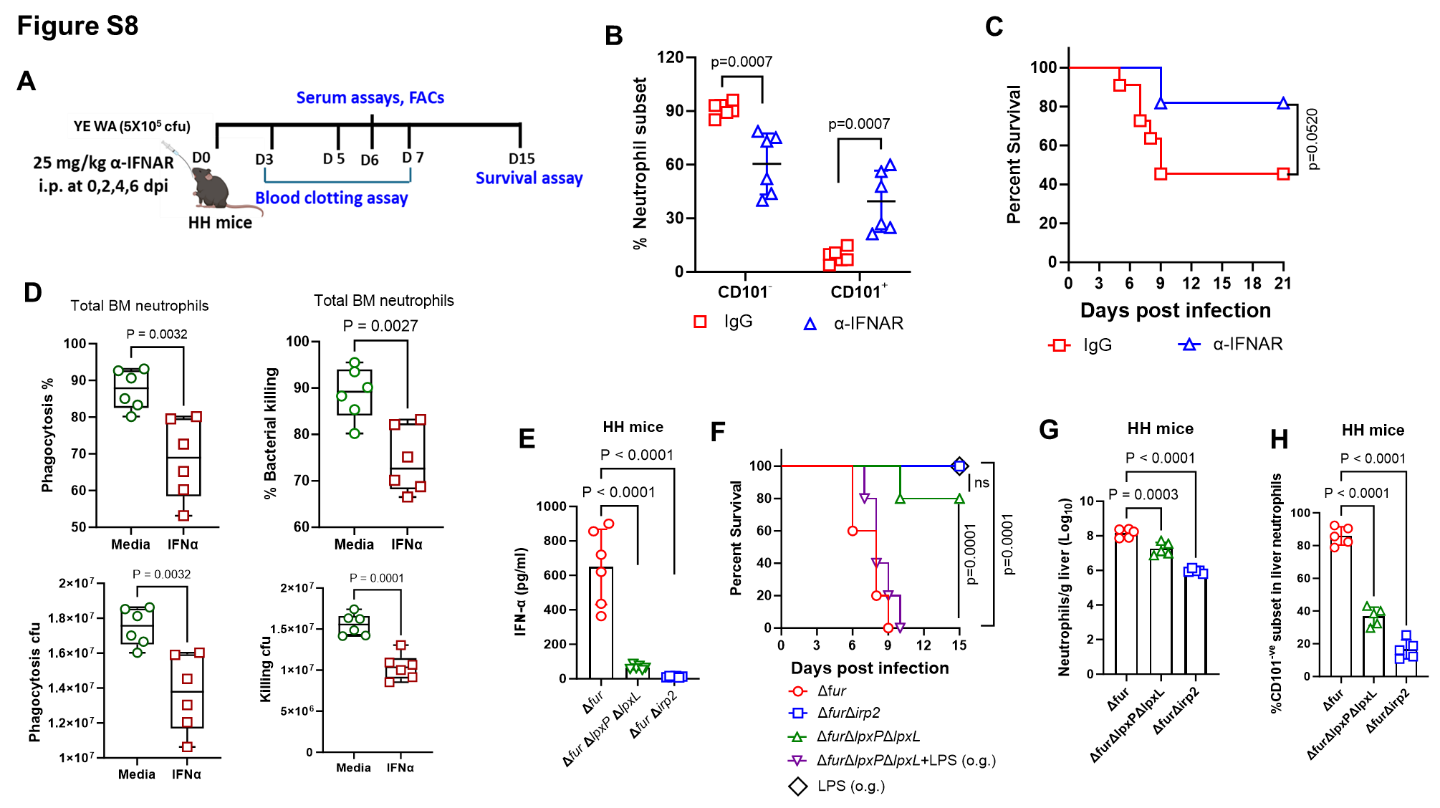


**Figure S8. Neutralization of IFNAR signaling in Ye WA infected HH mice and infection of HH mice with various Δ*fur* mutants.** (A) Schematic illustrating inhibition of IFNAR signaling via anti-IFNAR antibody administration in Ye WA infected HH mice. (B) Proportions of CD101⁺ and CD101⁻ neutrophils in the liver of HH mice treated with anti-IFNAR or IgG at 6 dpi. (C) Survival analysis of Ye WA infected HH mice treated with anti-IFNAR or IgG antibodies. (D) Phagocytic and bactericidal activity of total bone marrow neutrophil treated ex vivo with IFN-α. Total neutrophils were isolated from the bone marrow of HH mice, pretreated with IFN-α or media for 12 h, followed by infection with Δfur (MOI =5) for 2 h as described in methods. Bactericidal activity was calculated by comparing the CFU counts obtained after the bactericidal assay to those from the phagocytosis assay. (E) Quantification of IFN-α levels in liver homogenates of HH mice infected with the indicated bacterial strains (5×10^7^ CFU in 200 µl PBS) at 6 dpi. (F) Survival analysis of HH mice (n=5/group) infected with the indicated bacterial strains as mentioned above with/or without LPS (20 µg /mouse, oral gavage) as indicated. (G) Total neutrophil counts and (H) Proportions of CD101⁺ and CD101⁻ neutrophils in the liver of HH mice at 6 dpi. For all panels, unless otherwise mentioned, each symbol represents data from an individual mouse. Statistical analyses of comparisons of data among groups were performed with one-way ANOVA/univariate or two-way ANOVA with the Tukey post-hoc test. The log-rank (Mantel-Cox) test was used for survival analysis. Data are presented as the mean ± standard deviation (ns, no significance; * *P*< 0.05; ** *P*< 0.01; *** *P*< 0.001; **** *P*<0.0001).

References:

1. Das S, Saqib M, Meng RC, Chittur SV, Guan Z, Wan F, Sun W. 2022. Hemochromatosis drives acute lethal intestinal responses to hyperyersiniabactin-producing *Yersinia pseudotuberculosis*. Proc Natl Acad Sci U S A 119.

2. Weinberg GA. 1994. Iron chelators as therapeutic agents against *Pneumocystis carinii*. Antimicrob Agents Chemother 38:997-1003.
